# Early Steps in the Biosynthetic Pathway of Rishirilide B

**DOI:** 10.3390/molecules25081955

**Published:** 2020-04-23

**Authors:** Philipp Schwarzer, Olga Tsypik, Chijian Zuo, Ahmad Alali, Julia Wunsch-Palasis, Tanja Heitzler, Jana Derochefort, Mirjam Bernhardt, Xiaohui Yan, Thomas Paululat, Andreas Bechthold

**Affiliations:** 1 Institute for Pharmaceutical Biology and Biotechnology, Albert-Ludwigs-Universität, Stefan-Meier-Straße 19, 79104 Freiburg, Germany; Philipp-schwarzer@gmx.de (P.S.); olga.tsypik@pharmazie.uni-freiburg.de (O.T.); cheagien@hotmail.com (C.Z.); ahmad.alali@pharmazie.uni-freiburg.de (A.A.); Wujul@gmx.de (J.W.-P.); heitzler@gmx.de (T.H.); derochefortjana@aol.com (J.D.); mirjam.bernhardt@pharmazie.uni-freiburg.de (M.B.); XYan@scripps.edu (X.Y.); 2 Organic Chemsitry II, Universität Siegen, Adolf-Reichwein-Strasse 2, 57068 Siegen, Germany; paululat@chemie.uni-siegen.de

**Keywords:** rishirilide biosynthesis, gene deletion, streptomyces, oxygenase, ketoreductase, λ-Red/ET mediated recombination

## Abstract

The biological active compound rishirilide B is produced by *Streptomyces bottropensis*. The cosmid cos4 contains the complete rishirilide B biosynthesis gene cluster. Its heterologous expression in the host *Streptomyces albus* J1074 led to the production of rishirilide B as a major compound and to small amounts of rishirilide A, rishirilide D and lupinacidin A. In order to gain more insights into the biosynthesis, gene inactivation experiments and gene expression experiments were carried out. This study lays the focus on the functional elucidation of the genes involved in the early biosynthetic pathway. A total of eight genes were deleted and six gene cassettes were generated. Rishirilide production was not strongly affected by mutations in *rslO2, rslO6* and *rslH*. The deletion of *rslK4* and *rslO3* led to the formation of polyketides with novel structures. These results indicated that RslK4 and RslO3 are involved in the generation or selection of the starter unit for rishirilide biosynthesis. In the *rslO10* mutant strain, two novel compounds were detected, which were also produced by a strain containing solely the genes *rslK1*, *rslK2*, *rslK3*, *rslK4*, and *rslA*. *rslO1* and *rslO4* mutants predominately produce galvaquinones. Therefore, the ketoreductase RslO10 is involved in an early step of rishirilide biosynthesis and the oxygenases RslO1 and RslO4 are most probably acting on an anthracene moiety. This study led to the functional elucidation of several genes of the rishirilide pathway, including *rslK4*, which is involved in selecting the unusual starter unit for polyketide synthesis.

## 1. Introduction

**Figure 1:**
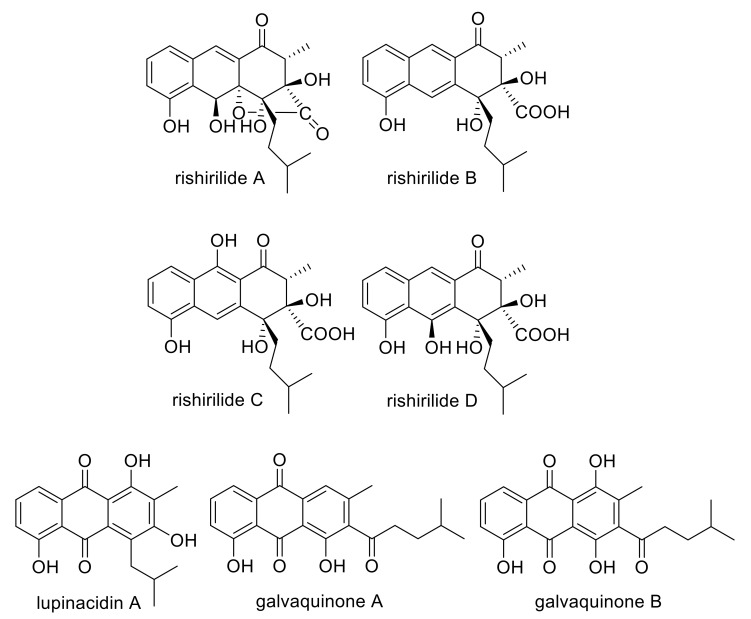
Structures of rishirilide A, B, C and D, lupinacidin A, galvaquinone A and galvaquinone B.

Polyketides are biosynthesized by polyketide synthases (PKS), which share striking similarities with fatty acid synthases. Of the three known PKS classes, the iterative type II PKS is relevant to our study. Type II polyketide biosynthesis starts by loading an α-carboxylated precursor (starter unit), normally acetate, onto an acyl carrier protein (ACP). The starter unit is subsequently transferred to the active site of a ketosynthase (KS) and then undergoes iterative elongation using malonyl-coenzyme A (CoA) as extender units to afford a nascent poly-β-keto chain. However, non-acetate starter units have been identified in type II polyketide biosynthesis as well. In these cases, a second catalytic module, the initiation module, can be found, consisting of a ketosynthase, a malonyl CoA:ACP transferase and an acyl carrier protein (ACP). The initiation module selects the starter unit and catalyzes the first elongation of the growing polyketide, before the molecule is loaded onto the minPKS for further elongation reactions. Additional enzymes, like ketoreductases, cyclases and aromatases complement the minPKS and determine the folding of the respective polyketide [1]. The aromatic polyketide rishirilide B (Figure 1) was first discovered by Iwaki et al. in 1984. Rishirilide B was isolated from *Streptomyces rishiriensis* OFR-1056 in the course of screening for new α2-macroglobulin inhibitors [2]. This compound was also described as a product of *S. olivaceus* SCSIO T05, which also produced rishirilide C, lupinacidin A and galvaquinone A and B [3] (Figure 1). The biosynthetic gene cluster of this strain responsible for the formation of all four compounds was cloned and biosynthetic studies were performed [3]. *S. bottropensis* (formerly *S. sp. Gö C4/4*) also produces the compounds rishirilide A, B, D and lupinacidin A (Figure 1). The corresponding rishirilide biosynthetic gene cluster was cloned on a cosmid (cos4), which was subsequently expressed in the heterologous host *S. albus* J1074. Rishirilide B was produced as a major compound. Moreover, rishirilide A, D and lupinacidin A were also produced in small amounts [3] (Figure 1). A rishirilide biosynthetic gene cluster was also cloned on a cosmid (cos4) (Figure S1) from *S. bottropensis* (formerly *S. sp. Gö C4/4*), which is the producer of rishirilide A, B and D and lupinacidin A (Figure 1). The cluster was heterologously expressed in *S. albus* J1074, resulting in the production of rishirilide B as the major product and of rishirilide A, rishirilide D and lupinacidin in a small amount [4] (Figure 1). The biosynthesis of rishirilide B was investigated by feeding experiments with different ^13^C labelled precursors. The NMR spectroscopic analysis of labelled compounds demonstrated that the tricyclic backbone of rishirilide B is a polyketide synthesized from nine acetate units. One of the acetate units is decarboxylated, resulting in a methyl group. The origin of the starter unit was determined to be isobutyrate [5]. Gene deletion experiments and in vitro enzyme studies were employed in *S. bottropensis* to identify key biosynthetic intermediates and expose intricate redox tailoring steps for the formation of rishirilides A, B, D and lupinacidin A. First, the epoxide-moiety of an advanced polycyclic intermediate is opened reductively by RslO5 to form an alcohol. Subsequently, the flavin monooxygenase RslO9 oxidatively rearranges the carbon backbone, presumably via lactone-forming Baeyer–Villiger oxidation and subsequent intramolecular aldol condensation. While RslO9 can further convert the rearranged intermediate to rishirilide D and lupinacidin A, an additional ketoreductase RslO8 is required for the formation of the main products rishirilide A and rishirilide B [6]. In this work, we have generated further mutants in order to gain insights into the early steps of rishirilide B biosynthesis and the role of the biosynthetic genes involved. Notably, we have constructed several cosmids, containing deletions in different genes (*rslK4, rslO1, rslO2, rslO3, rslO4, rslO6, rslO10 and rslH*). The heterologous expression of these mutated cosmids in *S. albus* J1074 has resulted in the identification of novel compounds. Based on the structure of these compounds, the function of RslK4, RslO3 and RslO10 was deduced. Furthermore, we have heterologously expressed genes from the rishirilid gene cluster in *S. albus* J1074 J1074 (*rslK1*, *rslK2*, *rslK3*, *rslK4*, *rslA*, (minPKS genes), as well as two genes, involved in the selection of the starter unit. We analyzed the natural compound production after overexpression of the min polyketidesynthase (PKS) genes in the presence or absence of several genes, including genes encoding the cyclases RslC1, RslC2 and RslC3, the primary ketoreductase RslO10 and the putative 3-oxoacyl-ACP reductase RslO3. A HPLC/ESI-MS analysis revealed that the emerging compounds were similar to the compounds of the gene deletion experiments, which provides us important information about rishirilide B biosynthesis.

## 2. Results

### 2.1. Overexpression of The Regulatory Genes rslR1, rslR2 and rslR3 Led to An Increased Rishirilide Production

Rishirilide biosynthesis is controlled by four regulatory genes (*rslR1*, *rslR2*, *rslR3* and *rslR4*) that belong to the family of SARP-regulatory genes (*rslR1* and *rslR2*), the family of LAL regulatory genes (*rslR3*) and the family of MarR transcriptional regulatory genes (*rslR4*), respectively [4]. We observed that *S. albus* J1074 containing cos4 and pUWL-H-*rslR1 + rslR2 + rslR3* (pUWLR1R2R3) produced more rishirilide B than *S. albus* J1074 containing only cos4. Therefore, pUWLR1R2R3 was always introduced into *S. albus* J1074. This strain was used as a host for further experiments.

### 2.2. Rishirilide Production Is Not Strongly Affected by Deletions of rslO2, rslO6, and rslH. 

Flavin-dependent oxidoreductases often require the reduced cofactor FMN for oxygen transfer [7]. NAD(P)H-flavin-dependent reductases are responsible for the allocation of reduced FMN. This group of enzymes catalyzes the reduction of FMN by NAD(P)H. The transfer of reduced FMN to the consuming oxidoreductase can occur by free diffusion processes, or directly by protein-protein interactions. *S. albus*::cos4∆O2 *x* pUWLR1R2R3 still produces rishirilide B (Figure S2), but also accumulates small amounts of compounds with rishirilide-like UV spectra. The predicted RslO2 function is flavin reduction and the reduced flavin can be consumed by the cluster encoded luciferase, like monooxygenases RslO1 and RslO6. Our results indicate that in the absence of RslO2, its function can be substituted by a Flavin reductase, encoded in the genome of *S. albus* J1074. Flavin dependent monooxygenases represent a special group of tailoring enzymes [8]. They catalyze the activation of molecular oxygen in the presence of a reduced flavin-cofactor, leading to the incorporation of oxygen into a molecule. *S. albus*::cos4∆O6 *x* pUWLR1R2R3 still produces rishirilide B (Figure S2), indicating that the flavin-dependent oxidoreductase RslO6 is not essential for rishirilide B production. Additionally, 6deletion of *rslH*, putatively encoding an amidohydrolase [9], also did not influence rishirilide B production (Figure S2), indicating that the gene does not belong to the cluster or that RslH activity is also not essential for rishirilide production.

### 2.3. Deletion of rslK4 Leads to the Formation of Derivatives with Alterations in the Side Chain, Indicating That RslK4 Is Involved in Selecting the Starter Unit for the PKS

**Figure 2:**
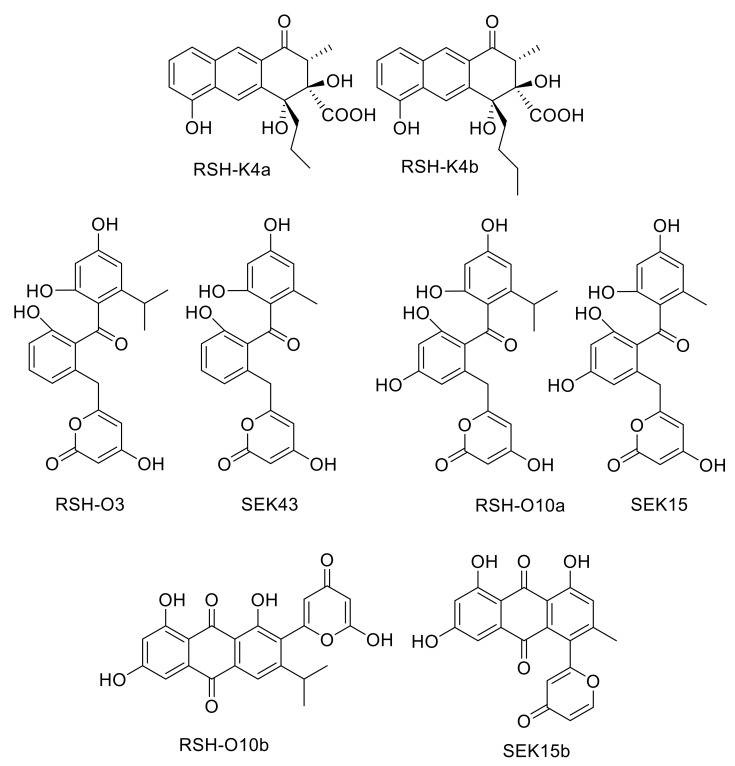
Compounds produced by strains generated during this study (RSH-K4a, putative structure of RSH-K4b, RSH-O3, RSH-O10a, RSH-O10b) and compounds known from the literature (SEK43, SEK15 and SEK15b [18]).

Acetyl-CoA, formed by decarboxylation of an activated malonyl unit, serves as the starter unit for the subsequent chain elongation in most aromatic polyketides. However, many deviant starter units are known, that are derived from malonate, benzoate, salicylate, or even amino acids like l-valine. [10,11,12] Unlike acetyl-CoA primed formations, the biosynthesis and attachment of unusual starter units often involves additional proteins that select, process and provide the starter units before transferring it to the minPKS for elongation. In rishirilide B biosynthesis, isobutyryl-CoA serves as the starter unit. This compound arises from the catabolism of l-valine and first gets converted to isobutyryl-CoA and finally to isohexanoyl-CoA, which is then loaded to the KS_α_/KS_β_ heterodimer of the PKS. The rishirilide biosynthetic gene cluster contains *rslK4*. BlastP analysis revealed the similarity of RslK4 to numerous KS III type ketosynthases, such as AknE2 from the aclacinomycin pathway [13,14], DpsC from the daunorubicin pathway [15] and ZhuH from the R1128 pathway [16,17]. AknE2 (DpsC), together with AknF (DpsD), are considered to be responsible for the selection of the propionate starter unit and the initiation of aclacinomycin (daunorubicin) biosynthesis. ZhuH encodes the KSIII in the initiation module of R1128 biosynthesis that, together with ZhuC (acyltransferase, AT), ZhuG (acyl carrier protein, ACP_P_) and endogenous fatty acid enzymes (ketoreductase, dehydratase and enoyl reductase), affect the generation of different acyl-ACP starter units. Thus, RslK4 might be involved in selecting the starter unit of the PKS during rishirilide biosynthesis. The cultivation of *S. albus*::cos4∆*rslK4 x* pUWLR1R2R3 resulted in the production of rishirilide B and several other compounds (Figure S2). Two of these compounds were named RSH-K4a and RSH-K4b (Figure 2). The structure of RSH-K4a was elucidated via mass spectrometry and NMR spectroscopy. The RSH-K4b structure was proposed, due to the similarity of RSH-K4a and RSH-K4b in their UV and mass spectra.

### 2.4. Deletion of rslO3 Leads to the Formation of A Shunt Product, Which Is Similar to SEK43, Indicating That RslO3 Is Involved in the Formation of the Starter Unit for the PKS

**Figure 3:**
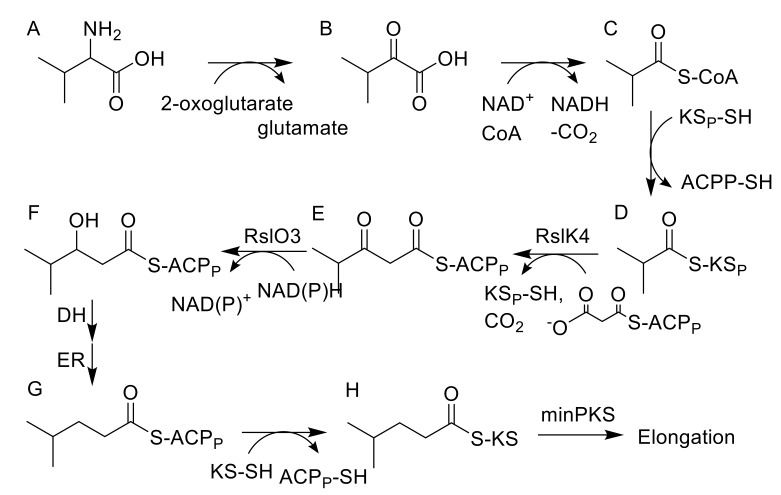
Proposed biosynthesis of the starter unit for rishirilide biosynthesis. (**A**) L-valine; (**B**) 2-ketoisovaleric acid; (**C**) isobutyryl-CoA; (**D**) isobutyryl-KS_P_; (**E**) 3-oxo-isohexanoyl-ACP_P_; (**F**) 3-hydroxy-ACP_P_ (**G**) isohexanoyl-ACP_P_; (**H**) isohexanoyl-KS.

BlastP analysis revealed high similarities of RslO3 to the 3-oxoacyl ACP reductase FabG from *Streptococcus pneumoniae* and SsfK from *S*. sp. SF2575 [19,20]. The conversion of l-valine to isohexanoyl-ACP, which is loaded to the KS_α_/KS_β_ heterodimer of the minPKS for further elongation, requires the formation of 3-hydroxy-ACP from 3-oxo-isohexanoyl-ACP (Figure 3) and this biosynthetic step might be catalyzed by RslO3. In *S. albus*::cos4∆*rslO3 x* pUWLR1R2R3, no rishirilide B production was observed. Instead, the strain produced a high amount of RSH-O3 (Figure 2).

### 2.5. Deletion of rslO10 Revealed the Function of RslO10 as Early Acting Ketoreductase

The first ring cyclization is—at least partly—controlled by the PKS that determines the initial region-specific folding and the cyclization pattern of nascent polyketides. In addition, the presence or absence of a site specific primary ketoreductase has a major impact on the first ring cyclization of type II PKS [21,22]. In the absence of a ketoreductase, a first ring cyclization between C9–C14 is often observed. A ‘reducing’ PKS associated with the presence of a ketoreductase often leads to a first ring cyclisation between C7–C12. The cyclization is initiated by reducing the carbonyl C-9 to a secondary alcohol. BlastP analysis revealed the presence of the ketoreductase gene *rslO10* in the rishirilide gene cluster. RslO10 shares high similarities with AknA, involved in aclacinomycin biosynthesis, and MsnO11, involved in mensacarcin biosynthesis. Both represent primary C-9 ketoreductases in their respective biosynthetic pathways [13,23]. In *S. albus*::cos4∆*rslO10 x* pUWLR1R2R3, two new shunt products, RSH-O10a and RSH-O10b (Figure 2), were accumulating in the mutant strain (Figure S2). RSH-O10a and RSHO10b represent decaketides that result from spontaneous cyclization of the highly reactive polyketide chain.

### 2.6. Expression of Gene Cassettes in S. albus J1074

In order to verify our results, we constructed gene cassettes for the coexpression of a few of the genes of the rishirilide gene cluster (Table 1, Figure S5). Plasmids were introduced into *S. albus* (Figure S2) containing pUWLR1R2R3. As expected, the expression of construct 1 containing the minimal PKS genes resulted in the formation of RSH-O10a and RSH-O10b. The same compounds were produced when all three cyclase genes were coexpressed with the minimal PKS genes (construct 2). Interestingly, when *rslO10* was coexpressed with the minimal PKS genes (construct 3), beside RSH-O10a, RSH-O3 was produced. This was also the case in a strain containing the minimal PKS genes, *rslO10* and the cyclase genes (construct 4). When *rslO3* was coexpressed with *rslO10* (construct 5 and 6), RSH-O3 was not produced. Only the expression of construct 6 led to the formation of tiny amounts of a new compound. Unfortunately, the structure of this compound could not be determined.

### 2.7. Galvaquinone A Is Produced by Mutants Lacking RslO1

The rishirilide biosynthetic gene cluster contains two luciferase-like monooxygenase genes [4]. Inactivation of *rslO6* did not influence rishirilide production as described above. In contrast, the inactivation of *rslO1* resulted in the formation of galvaquinone B (Figure 1 and Figure S2) as a major compound. The structure of galvaquinone B was elucidated via mass spectrometry and NMR spectroscopy (see below). 

### 2.8. Galvaquinone A and B Are Produced by Mutants Lacking rslO4

BlastP analysis identified RslO4 as a member of the antibiotic biosynthesis monooxygenase (ABM) superfamily. RslO4 shares high homology with SnoaB from *S. nogalater* [24], ActVA-Orf6 from *S. oelicolor* [25], as well as TcmH [26], which is involved in tetracenomycin biosynthesis in *S. glaucescens*. TcmH was the first characterized quinone—forming monooxygenase in Streptomyces. It catalyzes the conversion of the naphthacenone tetracenomycin F1 into 5,12-naphthacenequinone compound D3. ActVA-Orf6 converts 6-6-deoxydihydrokalafungin (6-DDHK) to dihydrokalafungin (DHK) during actinorhodin biosynthesis, whereas SnoaB catalyzes the conversion of 12-deoxynogalonic acid to nogalonic acid during nogalamycin biosynthesis. Common to all reactions is the addition of a keto moiety in the middle ring of the anthracene derivative without any assistance of prosthetic groups, metal ions and cofactors. To understand the function of RslO4 for rishirilide biosynthesis, a gene deletion of *rslO4* on cos4 was performed. A heterologous gene cluster expression was carried out, resulting in galvaquinone A and galvaquinone B production (Figure 1 and Figure S2). Both compounds were elucidated via mass spectrometry and NMR spectroscopy.

## 3. Discussion

Rishirilides are polyketides produced by several Streptomyces strains. Gene clusters responsible for their biosynthesis have been cloned from *S. bottropensis* [4] and from *S. olivaceus* SCSIO T05 [3]. Both gene clusters are very similar with an identical arrangement of the genes. The biosynthesis of rishirilides was recently investigated by our group. We published the function of RslO5, which reductively ring opens the epoxide—moiety of an advanced polycyclic intermediate, the function of the flavin monooxygenase RslO9 catalyzing a Baeyer-Villiger oxidation and intramolecular aldol condensation and the function of the ketoreductase RslO8 [6]. In *S. olivaceus* SCSIO T05, besides the four regulatory genes, *rsdK2*, *rsdO1*, *rsdO2*, *rsdO6*, and *rsdH* were inactivated. Rishirilide biosynthesis was not affected in the *rsdO6* and *rsdH* mutants and it was blocked in the *rsdK2* and *rsdO1* mutants. Deletion of *rsdO2* led to the accumulation of galvaquinone A and B. 

In this study, we deleted *rslK4*, *rslO1*, *rslO2*, *rslO3*, *rslO4*, *rslO6*, *rslO10* and *rslH* (Table 2). Our studies show that RslO6 and RslH are not essential for rishirilide biosynthesis. In contrast, the deletion of *rslO1* led to the production of galvaquinone A, indicating that RslO1 is involved in rishirilide biosynthesis, but not at an early stage. Additionally, as the *rslO4* mutant also accumulated galvaquinones, we suggest that RslO4 is involved in rishirilide biosynthesis and it also acts before rearrangement by RslO9 occurs. Surprisingly, the deletion of *rslO2* did not affect rishirilide production, indicating that a NAD(P)H-flavin-dependent reductase from *S. albus* could restore RslO2 activity, or that the flavin dependent reductase RslO1 receives sufficient FMN for activity in the cell and does not necessarily need RslO2. 

Isobutyryl-S-CoA has been described as the starter unit in other actinomycetes systems, including tautomycin and R1128C [11,27]. In rishirilide B, biosynthesis isobutyryl-S-CoA serves as the starter unit as well. It arises from the catabolism of l-valine via transamination and decarboxylation [5]. BlastP analysis revealed the similarity of RslK4 to numerous KS III type ketosynthases, including ZhuH. In R1128 biosynthesis ZhuH, together with ZhuC (AT), ZhuG (ACP_P_) and endogenous fatty acid enzymes (ketoreductase, dehydratase and enoyl reductase), is responsible for the generation of different acyl-ACP starter units [17]. We propose that the activation of the starter unit in rishirilide B resembles R1128 biosynthesis. RslK4 and RslA select the starter unit and catalyze the condensation with malonyl-CoA, to form a 3-oxoacyl-ACP intermediate. This intermediate is further modified by a ketoreductase, dehydratase and enoyl reductase. Unlike R1128 biosynthesis, the ketoreductase is not borrowed from fatty acid synthesis, but encoded by RslO3, a putative 3-oxoacyl ACP reductase. The emerging isohexanoyl-ACP_P_ is subsequently loaded to the KS_α_/KS_β_ heterodimer of the minPKS for further elongation (Figure 3). The Δ*rslO3* mutant accumulates RSH-O3 (Figure 2), which resembles SEK43 [18]. SEK43 was produced by combinatorial biosynthesis mixing the tcm minPKS, a ketoreductase from actinorhodin (act) gene cluster and an aromatase from the griseusin (gris) gene cluster. We believe that polyketo-derivatives accumulating in the Δ*rslO3* mutant and in the SEK43 producing strain are very similar, reacting to nearly identical derivatives.

**Figure 4:**
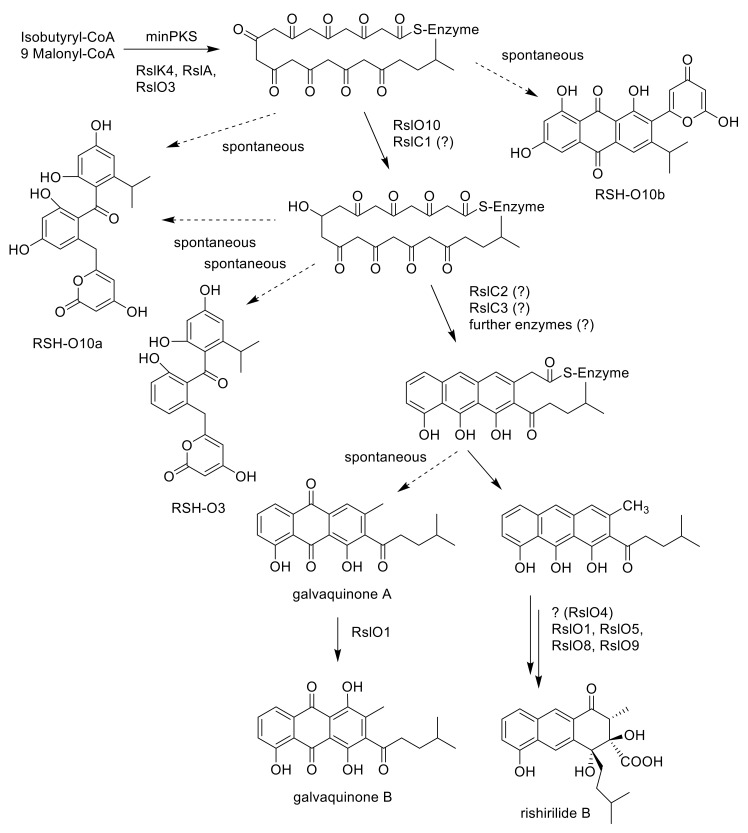
Proposed early steps in the biosynthetic pathway of rishirilide B. RSH-O10a, RSH-O10b. RSH-O3 and galvaquinones A and B are shunt products derived from an unstable polyketo-chain.

The importance of the ketoreductase RslO10 during the early steps of rishirilide B biosynthesis could be validated by a gene knockout of *rslO10* which leads to a complete breakdown of rishirilide B production; but two new intermediates, RSH-O10a and RSH-O10b (Figure 3) are produced. RSH-O10a and RSH-O10b represent decaketides that result from spontaneous cyclization of the highly reactive polyketide chain. Moreover, it can be deduced that the first ring cyclization in rishirilide B occurs at C7–C12. The presence of RslO10 as well as a C7–C12 first ring cyclization is in accordance with a ‘reducing’ PKS system. Regarding first ring cyclization, RSH-O10a represents the ‘natural and correct folded’ (C7–C12) intermediate. It emerges even in the absence of RslO10 by spontaneous folding of the poly-β-keto chain. Concurrently, due to aberrant cyclization events in the absence of RslO10, RSH-O10b is produced. It represents an intermediate of a different first ring cyclization (C9–C14). RSH-O10a and RSH-O10b are produced in a ratio of 5:1 (Figure S2), indicating a certain specificity for the regiochemistry of the natural first ring cyclization. When genes located on construct 1 (and construct 2) were expressed in *S. albus x* pUWLR1R2R3 RSH-O10a and RSH-O10b were also produced. Comparable experiments were performed by McDaniel, et al. [18], in which the heterologous expression of the minPKS of the tetracenomycin (tcm) cluster in *S. coelicolor* led to the emergence of the two intermediates, SEK15 and SEK15b (Figure 2) [18]. SEK15 shares high similarity with RSH-O10a and SEK15b with RSH-O10b, respectively. Differences in the structures of RSH-O10 and SEK15 derivatives are related to different starter units. Differences in the location of the pyrone moiety in RSH-O10b and SEK15b might also be explained by the size of the different starter units influencing spontaneous cyclization. Expression of construct 3 and construct 4 resulted in the formation of RSH-O10a and RSH-O3 but not RSH-O10b, indicating that RslO10 clearly supports the correct folding. In both cases, RSH-O3 was generated as well, which disappeared when *rslO3* was coexpressed (constructs 5 and 6). Although a new compound in a very tiny amount was produced, we assume that for the formation of a correct folded anthracene derivative, further genes have to be coexpressed. In Figure 4 early steps of the biosynthetic pathway of rishirilide B is proposed. 

## 4. Materials and Methods 

### 4.1. General 

All antibiotics and medium components used in this study were purchased from Carl Roth GmbH & Co. KG and Sigma-Aldrich (St. Louis, MO, USA). Restriction enzymes, T4 DNA ligase, and NEBuilder^®^ HiFi DNA Assembly Master Mix were bought from NEB Biotechnology Co. Ltd. (Ipswich, MA, USA). Plasmid, gel purification and cycle-pure kits were acquired from Promega (Fitchburg, WI, USA). Primer synthesis and DNA sequencing were performed by Eurofins Co., Ltd. (Luxenburg, Luxenburg).

### 4.2. Strains, Plasmids and Culture Conditions

Knockout experiments were performed in *S. albus* J1074::cos4, a transformant, which harbors the whole rishirilide gene cluster. The expression of gene cassettes was performed in *S. albus* J1074 as well. All strains were grown in TSB media (CASO bouillon 30 g × L^–1^, Carl Roth, Karlsruhe, Germany), supplemented with appropriate antibiotics and incubated in shake flasks for 24 h at 28 °C.

### 4.3. Generation of Gene Deletion Mutants 

Gene deletion experiments were performed on cos4. The spectinomycin resistance cassette of pCDF-Duet-1 was amplified by the primer pair F_R_-H/R_R_-H for *rslH,* F_R_-K4/R_R_-K4 for *rslK4*, F_R_O1/R_R_-O1 for *rslO1*, F_R_-02/R_R_-O2 for *rslO2*, F_R_-O3/R_R_-O3 for *rslO3*, F_R_-O4/R_R_-O4 for *rslO4*, F_R_-O6/R_R_-O6 for *rslO6*, and F_R_-O10/R_R_-O10 for *rslO10* (primers are listed in Figure S3). The genes *rslK4*, *rslO10*, *rslO1, rslO3*, and *rslO4* were replaced in *E. coli* DHα *x* pBADαβγ using the Redirect^®^ technology [28]. The introduced resistance marker was excised by NheI restriction and relegation. In each case, PCR was used to verify gene deletion using primers listed in Figure S3. The resulting cosmids were integrated into *S. albus* J1074 by intergeneric conjugation. pUWL-H-R1R2R3 was introduced into the mutated strain. 

### 4.4. Complementation of S. albus::cos4∆H, S. albus::cos4∆K4, S. albus::cos4∆O1, S. albus::cos4∆O2, S. albus::cos4∆O3, S. albus::cos4∆O4, S. albus::cos4∆O6 and S. albus::cos4∆O10.

For complementation, the genes were amplified using cos4 as a template and primer pairs F-H/R-H for *rslH,* F-K4/R-K4 for *rslK4*, F-O1/R-O1 for rslO1, F-02/R-O2 for *rslO2*, F-O3/R-O3 for *rslO3*, F-O4/R-O4 for *rslO4*, F-O6/R-O6 for *rslO6*, and F-O10/R-O10 for *rslO10* (Figure S3). PCR products were introduced into pUWL-H [29]. Subsequently, they were introduced in the corresponding mutated *S. albus*::cos4 construct. Expression of the complementation constructs restored rishirilide B biosynthesis in each case.

### 4.5. Generation of Strains Containing Different Gene Cassettes

Maps of plasmids generated during this study are shown in Figure S5.

#### 4.5.1. *S. albus x* pUWL-H-R1R2R3 

All three genes were amplified by PCR using primer pairs F-R1/R-R1 for *rslR1*, F-R2/R-R2 for *rslR2*and F-R3/R-R3 for *rslR3* (Figure S3). PCR fragments were successively cloned into pBluescript SK (-). *RslR1* was cloned via ClaI and HindIII, *rslR2* via HindIII, and *rslR3* via EcoRI and SpeI. The gene cassette containing all three genes was inserted into pUWL-H via ClaI and SpeI. The final vector was introduced into various constructs by intergeneric conjugation.

#### 4.5.2. *S. albus* J1074 *x* pUWLR1R2R3 *x* construct 1

The minPKS genes (*rslK1*, *rslK2*, *rslK3*), as well as *rslK4* and *rslA*, are located side by side on cos4. This region is flanked by BamHI restriction sites. Restriction with BamHI led to a 6107 bps fragment including the five genes, which was purified and cloned into the cloning vector pBluescript SK (-). The fragment was excised by EcoRI and XbaI and cloned into the final vector pTESa [30]. The resulting plasmid was introduced into *S. albus* J1074 *x* pUWLR1R2R3 by intergeneric conjugation yielding *S. albus* J1074 *x* pUWLR1R2R3 *x* construct 1 (Table 1).

#### 4.5.3. *S. albus* J1074 *x* pUWLR1R2R3 *x* construct 2

To introduce all three cyclase genes, *rslC1, rslC2* and *rslC3* were amplified by PCR with the primer pairs F-C1/R-C1, F-C2/R-C2 and FC-3/RC-3, respectively. They were cloned into the cloning vector pBluescript SK (-), in the order *rslC3*, *rslC2*, *rslC1*, to obtain pBSK-rslC1C2C3. The fragment (containing *rslC1*, *rslC2* and *rslC3*) was amplified by PCR using F-C123 and R-C3 and cloned into the XbaI site of pTESa containing minPKS + *rslK4* and *rslA* (Table 1). The resulting plasmid was introduced into *S. albus* J1074, yielding *S. albus* J1074 *x* pUWLR1R2R3 *x* construct 2 by intergeneric conjugation (Table 1).

#### 4.5.4. *S. albus* J1074 *x* pUWLR1R2R3 *x* construct 3*, S. albus* J1074 *x* pUWLR1R2R3 x construct 4, *S. albus* J1074 *x* pUWLR1R2R3 *x* construct 5 and *S. albus* J1074 pUWLR1R2R3 *x* construct 6

The gene *rslO10* alone and together with *rslO3* was introduced into *S. albus x* pUWLR1R2R3 *x* construct 1 and *S. albus x* pUWLR1R2R3 *x* construct 2, respectively, using the integrative vector pTOSz [30]. Since pTESa and pTOSz share the same antibiotic resistance, the already integrated apramycin resistance cassette, introduced by pTESa, was excised using site-specific recombination to obtain a marker free construct [30]. The gene *rslO10* was amplified by PCR with the primer pair F-O10/R-O10. The gene was introduced into pTOSz via XbaI and ClaI. The resulting plasmid was introduced into *S. albus* J1074 *x* pUWLR1R2R3 *x* construct 1 and *S. albus* J1074 *x* pUWLR1R2R3 *x* construct 2 by intergeneric conjugation yielding *S. albus x* pUWLR1R2R3 *x* construct 3 and *S. albus x* pUWLR1R2R3 *x* construct 4. The gene *rslO3* was amplified by the primer pair F-O3/R-O3 and cloned into pBluescript SK (-) via SmaI. The gene was excised and cloned into pTOSz by EcoR and XbaI. Then, *rslO10* was amplified using the primer pair F-O10/R-O10 and cloned into the *rslO3* containing pBluescript SK (-) vector via SpeI. The genes *rslO3* and *rslO10* were excised by EcoRI and XbaI and cloned into pTOSz before introducing into *S. albus* J1074 *x* pUWLR1R2R3 *x* construct 1 and *S. albus* J1074 *x* pUWLR1R2R3 *x* construct 2 yielding *S. albus x* pUWLR1R2R3 *x* construct 5 and *S. albus x* pUWLR1R2R3 *x* construct 6 (Table 1).

### 4.6. Metabolite Analysis by HPLC-MS.

After cultivation in production media, the supernatant was extracted with the same volume of ethyl acetate, evaporated and dissolved in methanol. The methanol extract was directly used for LC-MS analysis on a Thermo Fisher LC/MSD TSQ Quantum Access Max machine. For the LC analysis, a Zorbax Eclipse Plus C18 column (1.8 mcm, 2.1 mm × 50 mm) from Agilent was used (mobile phase A: H_2_O and mobile phase B: acetonitrile, both with 0.5% CH_3_COOH). The solvents were delivered at 0.5 mL/min with the following gradient: 0 min 80% A, 0.5 min 80% A, 8 min 5% A, 9 min 5% A, 9.1 min 80% A, 11 min 80% A. The mass spectrometer was operated in negative ion ESI mode, with an ion-spray voltage of −2.5 kV, source temperature of 450 °C. The pressures of sheath and auxiliary gas (N_2_) were set to 25 and 5 (arbitrary units), respectively. The ion transfer tube was heated up to 320 °C. 

### 4.7. Production of RSH-K4a, RSH-O10a, RSH-O10b, RSH-O3 and Galvaquinones A and B 

RSH-K4a was produced by *S. albus*::cos4*∆*rslK4 *x* pUWLR1R2R3, RSH-O10a by *S. albus*::cos4∆*rslO10 x* pUWLR1R2R3, RSH-O10b by *S. albus x* pUWLR1R2R3 *x* construct 1, RSH-O3 by *S. albus*::cos4∆*rslO3 x* pUWLR1R2R3 and galvaquinone A and B by *S. albus*::cos4∆*rslO4 x* pUWLR1R2R3. The strains were inoculated in TSB media, supplemented with appropriate antibiotics in shake flasks at 28 °C. The production media was inoculated with a 24 h old preculture (1% (*v/v*)).

### 4.8. Isolation and Purification of RSH-K4a, RSH-O10a, RSH-O10b, RSH-O3 and Galvaquinone A and Galvaquinone B

RSH-K4a, RSH-O10a, RSH-O10b, RSH-O3 and galvaquinone A and B were produced and purified in a similar way. RSH-K4a was isolated from 10 L of culture broth of DNPM production media (Bacto Soytone 7.5 g/L, dry yeast 5 g/L, MOPS 21 g/L, pH 6.8); RSH-O10a and RSH-O3 were produced in 2 L DNPM media, RSH-O10b was produced in 3 L HA media (glucose 4 g/L, yeast extract 4 g/L, malt extract 10 g/L, pH 7.4), galvaquinone A was produced in 7.5 L SG^+^ media (glucose 20 g/L, soytone 10 g/L, CaCO_3_ 2 g/L, CoCl_2_ 1 mg/L, l-valine 0.1% (m/V), pH 7.2), whereas galvaquinone B was produced in 10 L SG^+^ media. All media were supplemented with appropriate antibiotics. Production was carried out in shake flasks at 28 °C for 4–5 days. The culture broth was centrifuged and the supernatant was adjusted to pH 4. The supernatant was extracted with equal amounts of EtOAc, which was subsequently evaporated to dryness. In deviation to this, galvaquinone A and B were extracted using Diaion-HP20 (Merck, Darmstadt, Germany). Therefore, 10 g/L Diaion-HP20 was added and the mixture was incubated for 2 h at 90 rpm. The Diaion-HP20 particles were collected and repeatedly extracted with 250 mL EtOAc. For all compounds, the extraction was followed by fractionation using solid phase extraction (Oasis^®^ HLB20 35 cc (6 g) LP Extraction Cartridge, Waters GmbH, Eschborn, Germany), with increasing methanol concentrations (10% increments). The compounds were eluted from the column at certain concentrations. RSH-O10b, RSH-O3 and galvaquinone A were directly further purified by preparative HPLC, whereas for RSH-K4a, RSH-O10a and RSH-O10b, an additional purification step by TLC (Kieselgel 60 F_254_ TLC plates-20 x 20 cm, 2 mm, Merck, Darmstadt, Germany) followed. CH_2_Cl_2_ and MeOH in a ratio of 9:1 and additionally 0.05% HAc were used as solvents. The bands were scratched off the plate and repeatedly extracted with MeOH. All compounds except RSH-O10b were purified by semi-preparative HPLC. RSH-K4a, RSH-O3, and galvaquinones A and B were purified using a Zorbax^®^ SB-C18 precolumn (5 µm, 9.4 × 20 mm) and a Zorbax^®^ SB-C18 main column (5 µm, 9.4 × 150 mm), coupled to a DAD UV detector (Agilent, 1100 series, Agilent Technologies, Waldbronn, Germany). The column was eluted at a flow rate of 2 mL·min^–1^. RSH-O10a was purified by semi preparative HPLC, equipped with a XBridge^®^ C18 precolumn (3.5 µm, 4.6 × 20 mm) and a XBridge^®^ C18 main column (3.5 µm, 4.6 × 100 mm), coupled to a PDA detector (Waters Corporation, Milford, CT, USA). The column was eluted at a flow rate of 0.5 mL·min^–1^. Finally, all samples were purified using a Sephadex^®^ LH20 (GE Healthcare GmbH, Solingen, Germany) packed column. The sample was separated in 1 mL MeOH fractions. The fractions containing the desired compound were merged and evaporated to dryness.

### 4.9. NMR Analysis of Isolated Compounds

^1^H and ^13^C NMR spectra were recorded on a Varian VNMR-S 600, equipped with a 3 mm triple resonance inverse and 3 mm broadband probes (600/150 MHz). NMR spectra of galvaquinone B were measured using a Jeol ECZ-500 (Jeol, Akishima, Japan), equipped with a 5 mm Royal probe (500/125 MHz). NMR spectra of RSH-K4a were recorded using a Bruker Avance II (Bruker, Billerica, MA, USA) 400, equipped with a 1.7 mm triple resonance inverse probe (400/100 MHz). Spectra were recorded in DMSO-d_6,_ CD_3_OD or Acetone-d_6_ at 25 °C or 35 °C. Residual solvent signals were used for referencing (DMSO-d_6_: δ_H_: 2.50 ppm, δ_C_: 39.5 ppm; CD_3_OD: δ_H_: 3.30 ppm, δ_C_: 49.0 ppm; Acetone-d_6_: δ_H_: 2.05 ppm, δ_C_: 29.8 ppm). Solvents were purchased from Deutero (Kastellaun, Germany). NMR data are given in Supplementary Material (Figure S4).

## Figures and Tables

**Table 1:** Strains generated during this study and major compounds produced by the strains.

Description of Mutant Strains	Compound Produced by the Strain
*S. albus*::cos4 *x* pUWLR1R2R3	Rishirilide B
*S. albus*::cos4∆*rslO2 x* pUWLR1R2R3	Rishirilide B
*S. albus*::cos4∆*rslO6 x* pUWLR1R2R3	Rishirilide B
*S. albus*::cos4∆*rslH x* pUWLR1R2R3	Rishirilide B
*S. albus*::cos4∆*rslK4 x* pUWLR1R2R3	RSH-K4a, RSH-K4b and rishirilide B
*S. albus*::cos4∆*rslO3 x* pUWLR1R2R3	RSH-O3
*S. albus*::cos4∆*rslO10 x* pUWLR1R2R3	RSH-O10a and RSH-O10b
S. albus J1074 *x* pUWLR1R2R3 *x* construct 1 (construct 1: *rslK1*, *rslK2*, *rslK3*, *rslK4*, *rslA*)	RSH-O10a and RSH-O10b
S. albus J1074 *x* pUWLR1R2R3 *x* construct 2 (construct 2: *rslK1*, *rslK2*, *rslK3*, *rslK4*, *rslA*, *rslC1*, *rslC2*, *rslC3*)	RSH-O10a and RSH-O10b
S. albus J1074 *x* pUWLR1R2R3 *x* construct 3 (construct 3: *rslK1*, *rslK2*, *rslK3*, *rslK4*, *rslA*, *rslO10*)	RSH-O10a and RSH-O3
S. albus J1074 *x* pUWLR1R2R3 *x* construct 4 (construct 4: *rslK1*, *rslK2*, *rslK3*, *rslK4*, *rslA*, *rslO10*, *rslC1*, *rslC2*, *rslC3*)	RSH-O10a and RSH-O3
S. albus J1074 *x* pUWLR1R2R3 *x* construct 5 (construct 5: *rslK1*, *rslK2*, *rslK3*, *rslK4*, *rslA*, *rslO10*, *rslO3*)	RSH-O10a
S. albus J1074 *x* pUWLR1R2R3 *x* construct 6 (construct 6: *rslK1*, *rslK2*, *rslK3*, *rslK4*, *rslA*, *rslO10*, *rslO3*, *rslC1*, *rslC2*, *rslC3*)	RSH-O10a
*S. albus*::cos4∆*rslO1 x* pUWLR1R2R3	Galvaquinone A
*S. albus*::cos4∆*rslO4 x* pUWLR1R2R3	Galvaquinone A and B

**Table 2:** Genes discussed in this study and their deduced function.

Analyzed Genes in This Study	Deduced Function
*rslK4*	ketosynthetase, selection of unusual starter unit
*rslO1*	luciferase-like monooxygenase, involved in late stage rishirilide biosynthesis, but before rearrangement by *rslO9*
*rslO2*	Flavin reductase
*rslO3*	3-oxoacyl ACP reductase, catalysis of l-valine to isohexanoyl-ACP conversion for formation of the starter unit
*rslO4*	antibiotic biosynthesis monooxygenase, involved in late stage rishirilide biosynthesis, but before rearrangement by *rslO9*
*rslO6*	Flavin dependent oxidoreductase, not essential for rishirilide biosynthesis
*rslO10*	C9-ketoreductase, support of C7–C12 ring cyclisation
*rslH*	amidohydrolase, not essential for rishirilide biosynthesis

## Supplementary Materials

The following are available online: Figure S1: Biosynthetic gene cluster of rishirilide B, Figure S2: HPLC analysis of extracts of mutants obtained in this study, Figure S3: Primers used in this study, Figure S4, ^1^H and ^13^C NMR data, Figure S5, plasmids generated during this study.
