# Supplementary material for: Early Steps in the Biosynthetic Pathway of Rishirilide B
Source: Molecules. 2020 Apr 23;25(8):1955. doi: 10.3390/molecules25081955 (PMC7221717; doi:10.3390/molecules25081955)

SUPPLEMENTARY

Figure S1: Biosynthetic gene cluster of rishirilide B

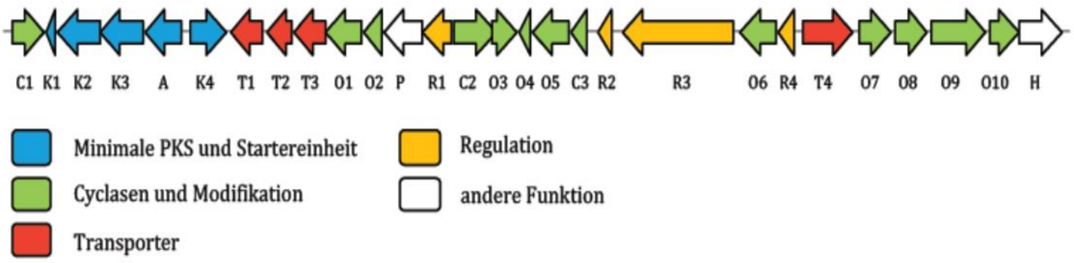

Figure S2: HPLC analysis of extracts of mutants obtained in this study. Rishirilide B (20,8 min) (1); RSH-O10a (17,3 min) (2); RSH-O10b (21,0 min) (4); RSH-O3 (18,5 min) (3); Galvaquinone A (26,5 min) (5); Galvaquinone B (27,8 min) (6); RSH-K4a (18,2 min) (7); RSH-K4b (19,6 min) (8). λ = 254 nm

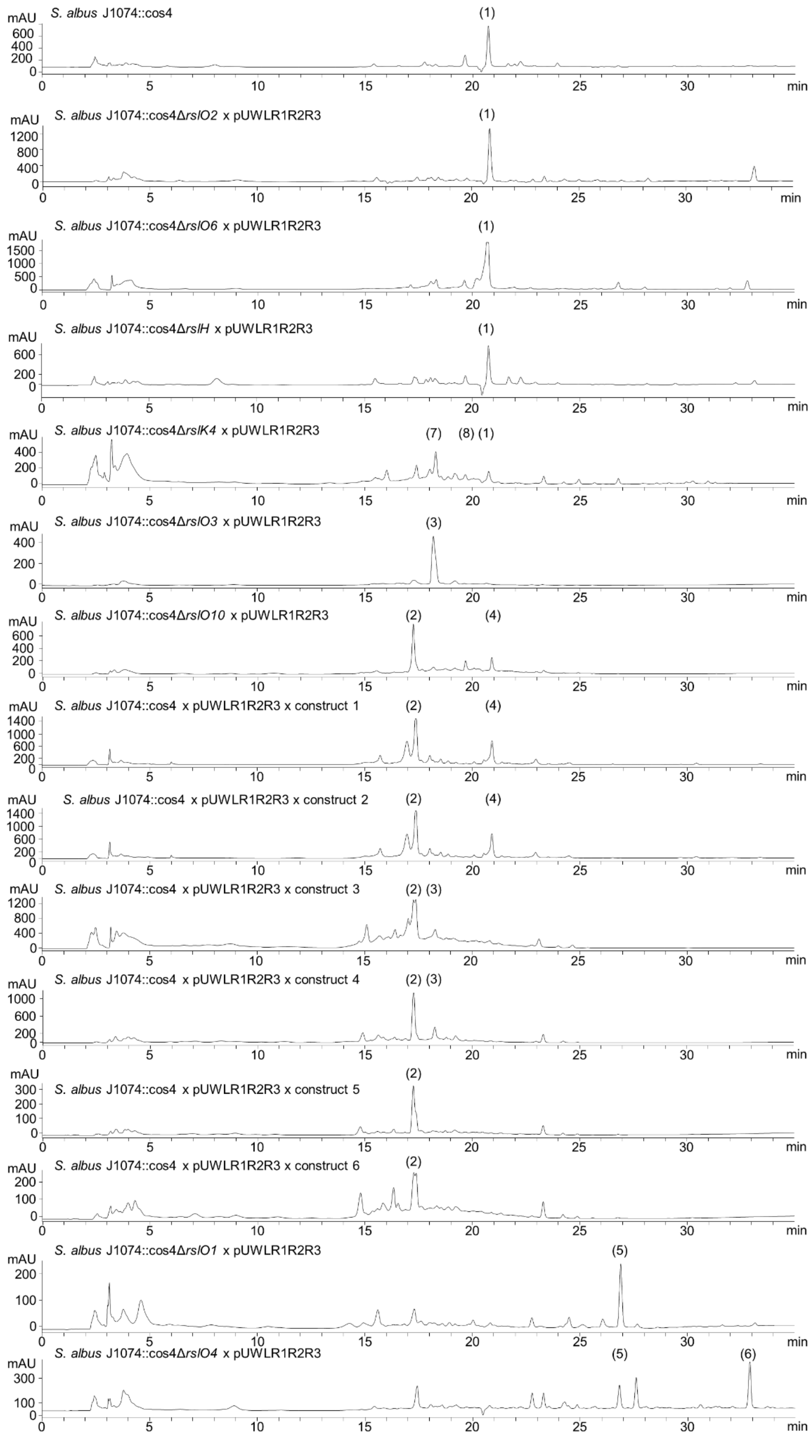

Figure S3: Primers used in this study

A: Primer used for Red/ET-mediated recombination

(F_R-H) 5’-TGCGGTGGTCTGGGCAACTACTGACGGTGGCCGGCCCATGGCTAGCGGAGCGTAGCGACCGAGTG -3’

(R_R-H) 5’- CTCGGGCCTCTCACGATGTTCGAGGGCCGCCCTCGCTCAGCTAGCGGCTATTTAACGACCCTGC -3’

(F_R-K4) 5’- TGGTTCCCCTGACGGCCGGTGAAAGGGCATCGGGACATGGCTAGCGGAGCGTAGCGACCGAGTG -3’

(R_R-K4) 5’- CCCGCTCGGCGCGCATGCGCCACCGGCGCGGCGCGGTCAGCTAGCGGCTATTTAACGACCCTGC -3’

(F_R-O1) 5‘-ACACCGTCCGTCCAGCCATCACATCGAGAGGACCCCATGGCTAGCGGAGCGTAGCGACCGAGTG -3’

(R_R-O1) 5‘-GCTCGGTGCGCAAGGAAACCATCGGCGGTCCGTCCCTCAGCTAGCGGCTATTTAACGACCCTGC -3’

(F_R-O2) 5‘-GAAAGCCTGAGCGGGCAAGGGAGGAGCTACAGACCCATGGCTAGCGGAGCGTAGCGACCGAGT -3’

(R_R-O2) 5‘-GGTGTGTGCGTCGCGGCCGGGGGCCGCGGCTGCCGGCTAAGCTAGCGGCTATTTAACGACCCTGC-3’

(F_R-O3) 5‘-CGGCGAAGTTGCGAAGCCGATGCTGTGGAGGAGAGATGGCTAGCGGAGCGTAGCGACCGAGTG -3’

(R_R-O3) 5‘-CACGCGCCGGCCCGCGATCGCCCGAGGGGACGCGCTCAGCTAGCGGCTATTTAACGACCCTG -3’

(F_R-O4) 5‘-CCACCCGCGTACGAACATCCACCCGAGGAGACCACCATGGCTAGCGGAGCGTAGCGACCGAGTG -3’

(R_R-O4) 5‘-GCGTGGTGTACGTGCCGGCCCGCGGTCCGCTCCGGCTCAGCTAGCGGCTATTTAACGACCCTGC -3’

(F_R-O6) 5‘-CCCCTCCTTTCGGCCCTGTCCGTGAAGGAGACCAGCGTGGCTAGCGGAGCGTAGCGACCGAGTG -3’

(R_R-O6) 5‘-GTTCCGGACATTCTTCGGCAGGCCGTCCGCTCCGCTTCAGCTAGCGGCTATTTAACGACCCTGC -3’

(F_R-O10) 5‘-CGCACCTCCCCGAACGTCATGGCGAAAGGATCCGCAATGGCTAGCGGAGCGTAGCGACCGAGT-3’

(R_R-O10) 5‘-CGCTCCTGGACTGGGGGCCTCATGGGCCGGCCACCGTCAGCTAGCGGCTATTTAACGACCCTGC -3’

B: Primer used to verify gene deletion

((F-Hver) 5’- ATTATCACCCGCGAGGTGCTG -3’

(R-Hver) 5’- AGTCGTTCGGGTTCACCATGC -3’

F-K4ver) 5‘-ATGAGGTTCGAGGAC -3’

(R-K4ver) 5‘-CGGATCCCGTCTCTC -3’

(F-O1ver) 5‘-CCAGCCACGGGCCGTCGGGCTTCACG -3’

(R-O1ver) 5‘-GTCCTCCTGGCAGCACAACCGCAGC -3’

(F-O2ver) 5‘-TGAAGACGGGCAGTACG -3’

(R-O2ver) 5‘-TGCTCGACTGGGAGATG -3’

(F-O3ver) 5‘-AGCTGATCCATCCCGGTGTG -3’

(R-O3ver) 5‘-TATCCGGTGCTCGTGGATGC -3’

(F-O4ver) 5‘-CGGGGTGACCGTGAACTGCG-3’

(R-O4ver) 5‘-TCGTCAACCTCGGCACCGGA- 3’

(F-O6)ver 5‘-GGAGACCAGCGTGAAACTG- 3’

(R-O6ver) 5‘-GTCCGCTCCGCTTCACGCG -3’

(F-O10ver) 5‘-GGAAGGAGCGTTCTCCACCG -3’

(R-O10ver) 5‘-CGGACGCGTACACCGAGAC -3’

C: Pimers used for the amplification of genes for complementation and coexpression experiments

(F-H) 5’- TAAAGCTTACGACCTCGCCGCCTCC -3’

(R-H) 5’- CATACTAGTCGTTCGGGTTCACCATGC -3’

(F-K4) 5‘-CCATCGATGGAGGCGGGACATGAGGTTCGAGGAC -3’

(R-K4) 5‘-CCACTAGTCATCCGGATCCCGTCTCTC -3’

(F-O1) 5‘-CTATCGATGGAGGACCCCATGAAGTTCGGC -3’

(R-O1) 5‘-GCACTAGTCCCTCAGTCGTTCGCTGC- 3’

(F-O2) 5‘-GCCAAGCTTATGGCGATCGATGACGAACTG -3’

(R-O2) 5‘-TATTGGATCCCGGCTAGTCGGCCGTCAC-3’

(F-O3) 5‘-ATAAGCTTTGCGCGAGATGGTC -3’

(R-O3) 5‘-ATACTAGTGCGAACCGCACCTG -3’

(F-O4) 5‘-CCATCGATAAGTCCGCCCCACGTACCCGC -3’

(R-O4) 5‘-GGACTAGTGCGGTCCGCTCCGGCTCAGA-3’

(F-O6) 5‘-TCATCGATGAAGGAGACCAGCGTGAAACTG -3’

(R-O6) 5‘-GGACTAGTCCGCTCCGCTTCACGCG -3’

(F-O10) 5‘-TATATCTGCAGCGGCCCGCGCACTGAAC -3’

(R-O10) 5‘-TACTGTCTAGAGACCGCGCCCAGGATG -3’

(FbO3) 5‘-ATATGCTCGGCGAAGTTGCG -3’

(RbO3) 5‘-ATATCCGGCACGTACACCAC -3’

(O10bF) 5‘-ATATCCCGAACGTCATGGC -3’

(O10bR) 5‘-TASTACGCTCCTGGACTGGG -3’

(F-R1) 5‘-CCCATCGATTCTCTTAAGGACCACGGAAGCCGCACC -3’

(R-R1) 5‘-GAACAGAAGCTTACGGCCGGCGCCGG -3’

(F-R2) 5‘-ATTCCGAAGCTTCAAGCCAGCCCTGGAGG -3’

(R-R2) 5‘-AACACTGCAGAGCTAGCGGGGGTCAGCCGGCC -3’

(F-R3) 5‘-TACGAATTCTCGCTAGCGGAGCGGACGGCCTC -3’

(R-R3) 5‘-GGACTAGTCACTGCTCCCGCCACCGT-3’

(F-C1) 5‘-CGGGGTACCTAACCCCCCACTTTT-3’

(R-C1) 5‘-CCAAGCTTCGTCGACCTGGTCAACTC-3’

(F-C2) 5‘-CCCAAGCTTATGGTGGATCCTCTCTTC-3’

(R-C2) 5‘-CCGGAATTCGCAGATCCATCTCTCCTCE-3’

(F-C3) 5‘-CGCGGATCCCACCTTCAGCACCGAAC-3’

(R-C3) 5‘-GCTCTAGATGGCCTCGCTGATGATGAGTCC-3’

(F-C123) 5‘-GCTCTAGAACCCCCCACTTTTCATGGAC-3’

Figure S4: ^{1}H and ^{13}C NMR and ESI-MS data

Figure S4a: NMR data of rishirilide B (600/150MHz, DMSO-d_6, 35 °C)

Pos.	δ_C [ppm]	δ_H (*J* Hz) [ppm]
1	197.1	
2	47.9	2.99 q (6.8)
3	83.6	
4	76.9	
4a	140.0	
5	153.0	
6	109.9	6.93 d (7.6)
7	126.3	7.28 dd (8.3, 7.6)
8	119.7	7.46 d (8.3)
8a	132.3	
9	125.7	8.29 s
9a	129.9	
10	119.6	8.28 s
10a	126.1	
11	35.0	11-H_a: 2.23 dt (13.1, 3.9)
		11-H_b: 1.61 ddd (13.3, 12.8, 4.7)
12	31.1	12-H_a: 1.38 m
		12-H_b: 0.78 m
13	27.8	1.30 m
14	22.4	0.66 d (6.5)
15	22.6	0.77 d (6.5)
16	174.0	
17	10.1	1.19 d (6.8)
OH		10.2 s br

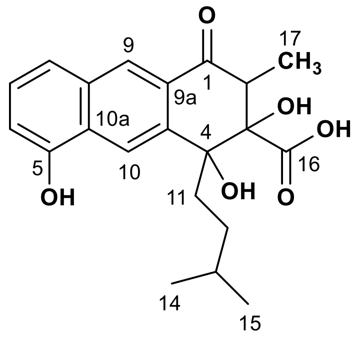

Figure S4b: NMR data of RSH-K4a (400/100MHz, CD_3OD, 25 °C)

Pos	δ_C [ppm]	δ_H (*J* Hz) [ppm]	COSY	HMBC
1	200.0			2-H, 9-H, 15-H_3
2	49.3[a]	3.05 q (6.8)	15-H_3	15-H_3
3	84.5			2-H, 15-H_3
4	78.9			10-H, 11-H_a
4a	140.8			
5	154.6			7-H, 10-H
6	111.2	6.89 d (8.0)	7-H	
7	127.6	7.28 t (8.0)	6-H, 8-H	
8	121.5	7.43 d (8.0)	7-H	9-H
8a	131.3			7-H
9	127.9	8.39 s		
9a	131.4			10-H
10	121.1	8.43 s		
10a	128.2			8-H
11	41.0	H_a: 2.30 m	11-H_b, 12-H_a, 12-H_b	
		H_b: 1.68 m	11-H_a, 12-H_a, 12-H_b	
12	17.2	H_a: 1.55 m	11-H_a, 11-H_b, 12-H_b, 13-H_3	
		H_b: 1.05 m	11-H_a, 11-H_b, 12-H_a, 13-H_3	
13	14.7	0.80 t (7.2)	12-Ha, 12-Hb	11-H_2, 12-H_2
14	178.0[b]			2-H
15	10.5	1.30 d (6.8)	2-H	2-H

[a]covered from solvent signal
[b]taken from HMBC

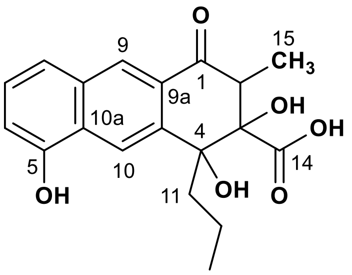

^{1}H NMR spectrum of RSH-K4a (400MHz, CD_3OD, 25 °C).

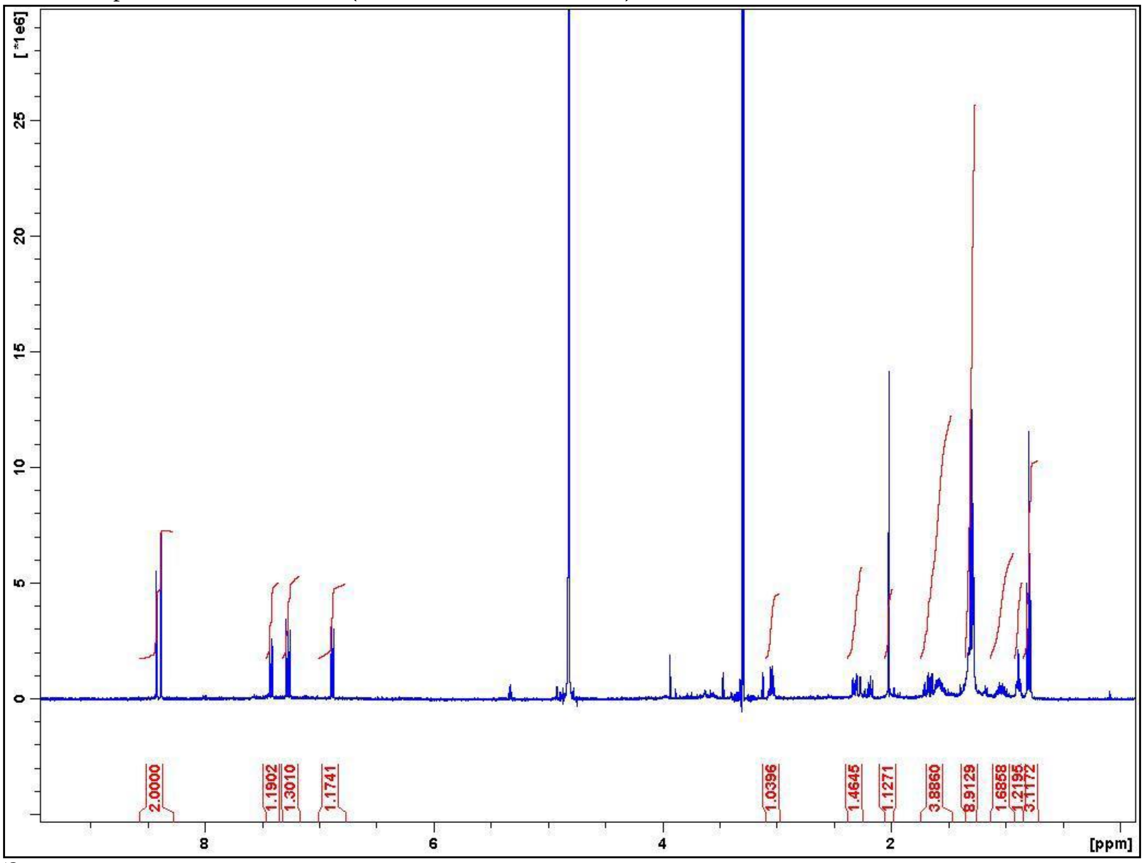

^{13}C NMR spectrum of RSH-K4a (100MHz, CD_3OD, 25 °C).

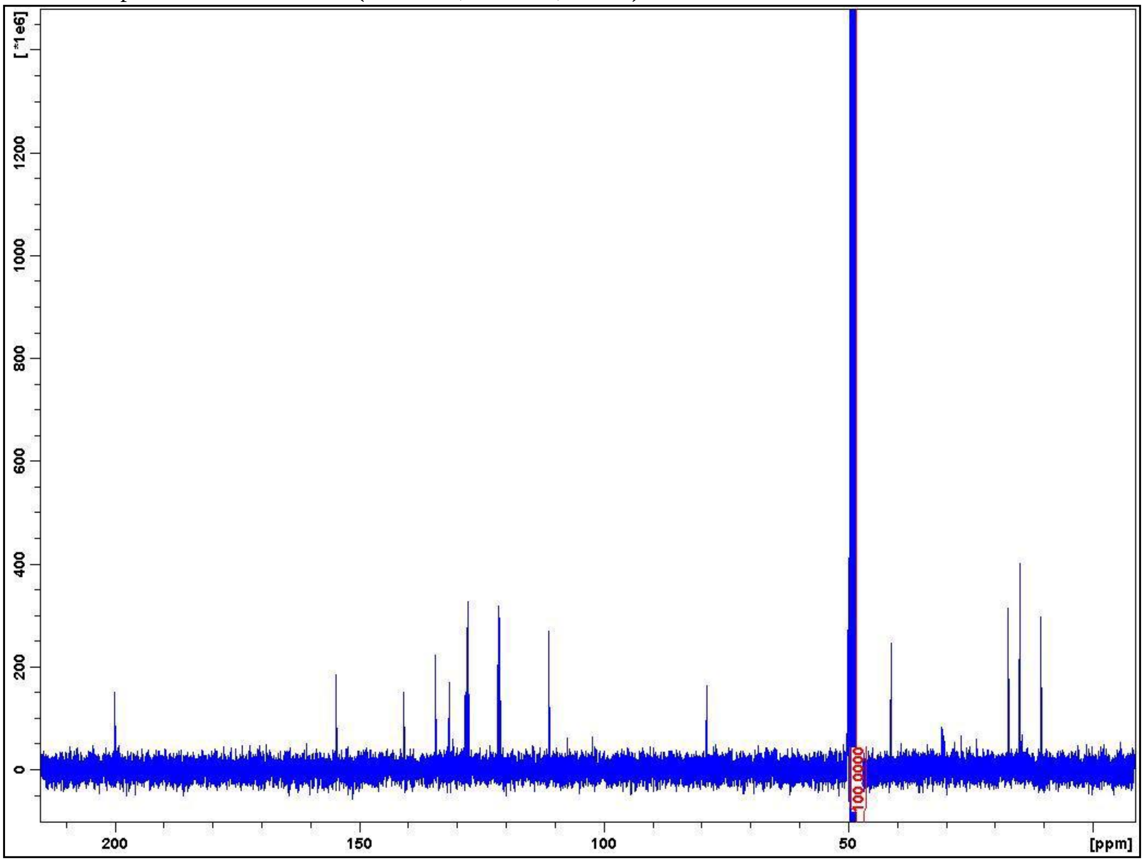

Figure S4c: NMR data of RSH-010a (600/150MHz, DMSO-d_6, 35 °C)

Pos.	δ_C [ppm]	δ_H (*J* Hz) [ppm]	COSY	HMBC
1	171.1			2-H, 4-H
2	88.1	5.09 d (2.0)	4-H	4-H
3	163.8			2-H
4	101.4	5.61 d (2.0)	2-H	2-H, 6-H_2
5	164.4			4-H, 6-H_2
6	36.7	3.55 s		4-H, 8-H
7	118.8			6-H_2, 8-H, 10-H
8	110.1	6.14 d (2.2)	10-H	6-H_2, 10-H
9	161.2			8-H, 10-H
10	101.6	6.21 d (2.2)	8-H	8-H
11	162.5			10-H
12	138.5			6-H_2
13	200.0			10-H, 16-H
14	120.5			16-H, 18-H, 20-H
15	156.4			16-H
16	100.1	6.09 d (2.0)	18-H	18-H
17	159.7			16-H, 18-H
18	104.0	6.20 d (2.0)	16-H	16-H, 20-H
19	149.2			20-H, 21-H_3, 22-H_3
20	29.7	2.77 hept (6.8)	21-H3, 22-H3	18-H, 21-H_3, 22-H_3
21	23.8	1.02 d (6.8)	20-H	20-H, ^{1}J, 22-H_3
22	23.8	1.02 d (6.8)	20-H	20-H, ^{1}J, 21-H3
OH		11.35 br s		

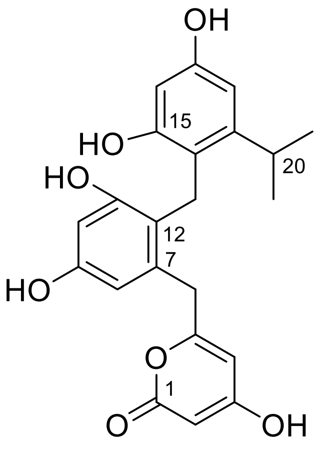

^{1}H NMR spectrum of RSH-O10a (600MHz, DMSO-d_6, 35 °C).

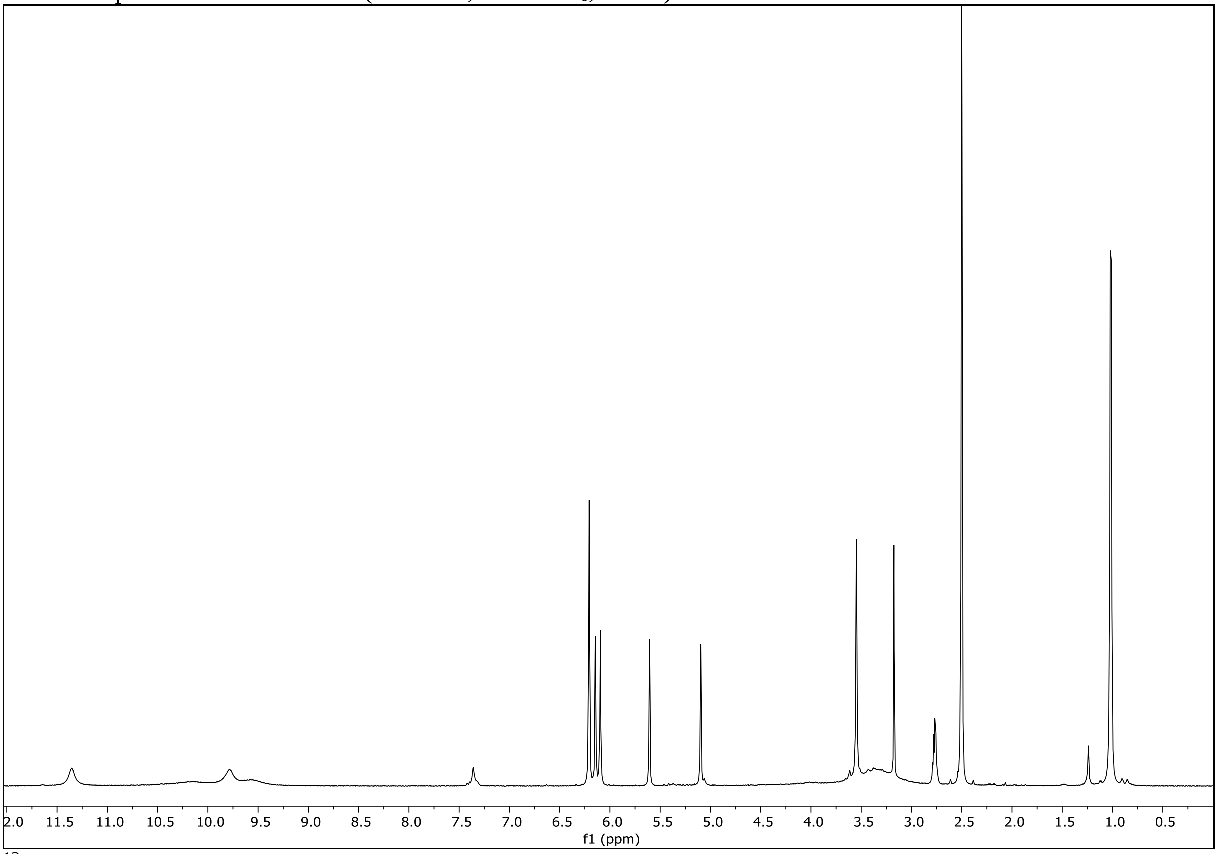

^{13}C NMR spectrum of RSH-O10a (150MHz, DMSO-d_6, 35 °C).

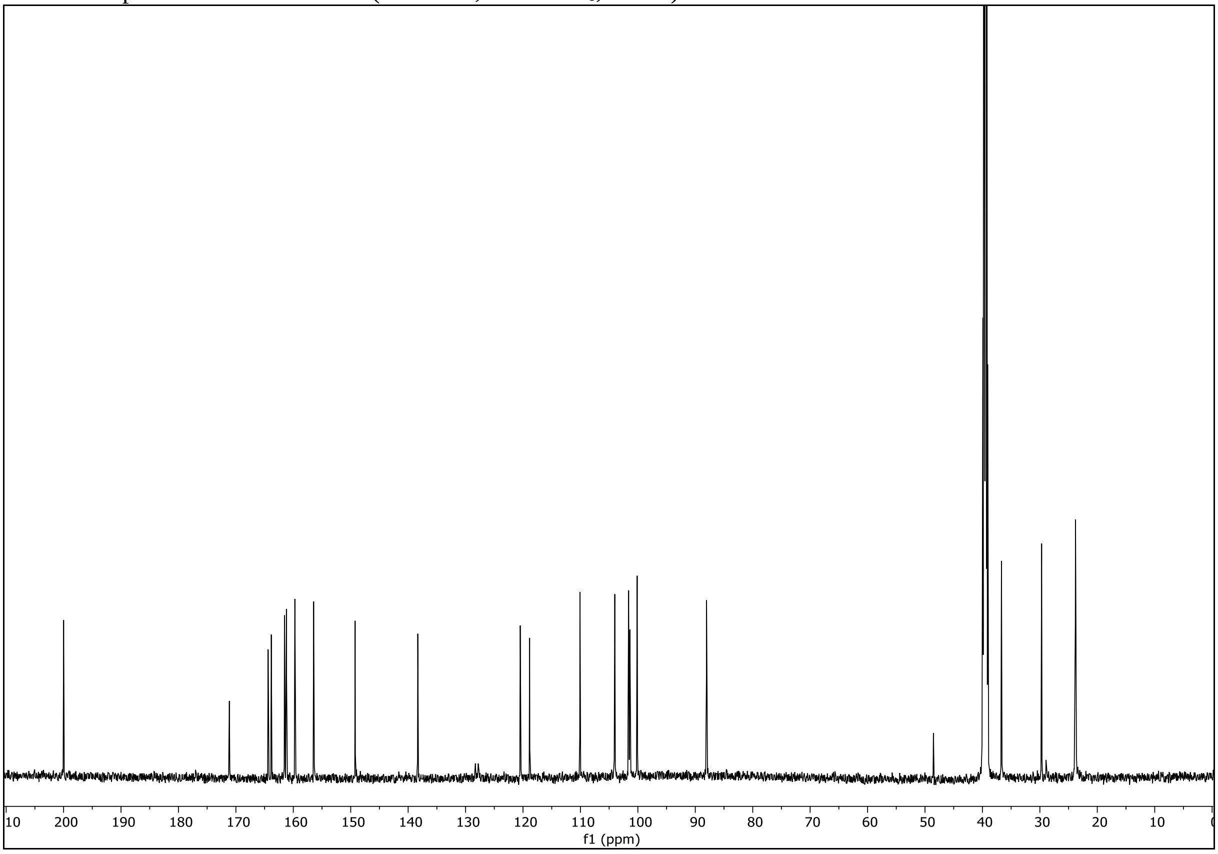

Figure S4d: NMR data of RSH-O10b (600/150MHz, DMSO-d_6, 35 °C)

Pos	δ_C [ppm]	δ_H (J Hz) [ppm]	COSY	HMBC [b]
1	159.4			-
2	126.7			4-H,12-H, 16-H
3	156.2			4-H, 16-H, 17-H3, 18-H_3
4	115.6	7.71 s		16-H
4a	134.9[a]			-
5	109.7	7.13 d (2.3)	7-H	7-H
6	164.6			7-H
7	108.0	6.55 d (2.3)	5-H	5-H
8	166.8			7-H
8a	108.4			7-H
9	188.5			(4-H)
9a	113.8			4-H
10	181.1			4-H, 5-H
10a	133.8[a]			-
11	155.4			12-H
12	106.1	6.19 d (1.9)	14-H	14-H
13	171.3			-
14	89.1	5.26 d (1.9)	12-H	12-H
15	163.9			14-H
16	30.8	2.99 hept (6.9)	17-H_3, 18-H_3	4-H
17	22.9	1.24 d (6.9)	16-H	16-H, 18-H_3
18	22.9	1.24 d (6.9)	16-H	16-H, 17-H_3
OH		12.59 br s		
		11.89 br s		

[a]signals exchangeable

[a]weak signals in brackets

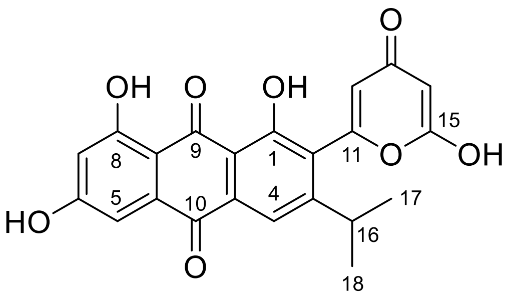

^{1}H NMR spectrum of RSH-O10b (600MHz, DMSO-d_6, 35 °C).

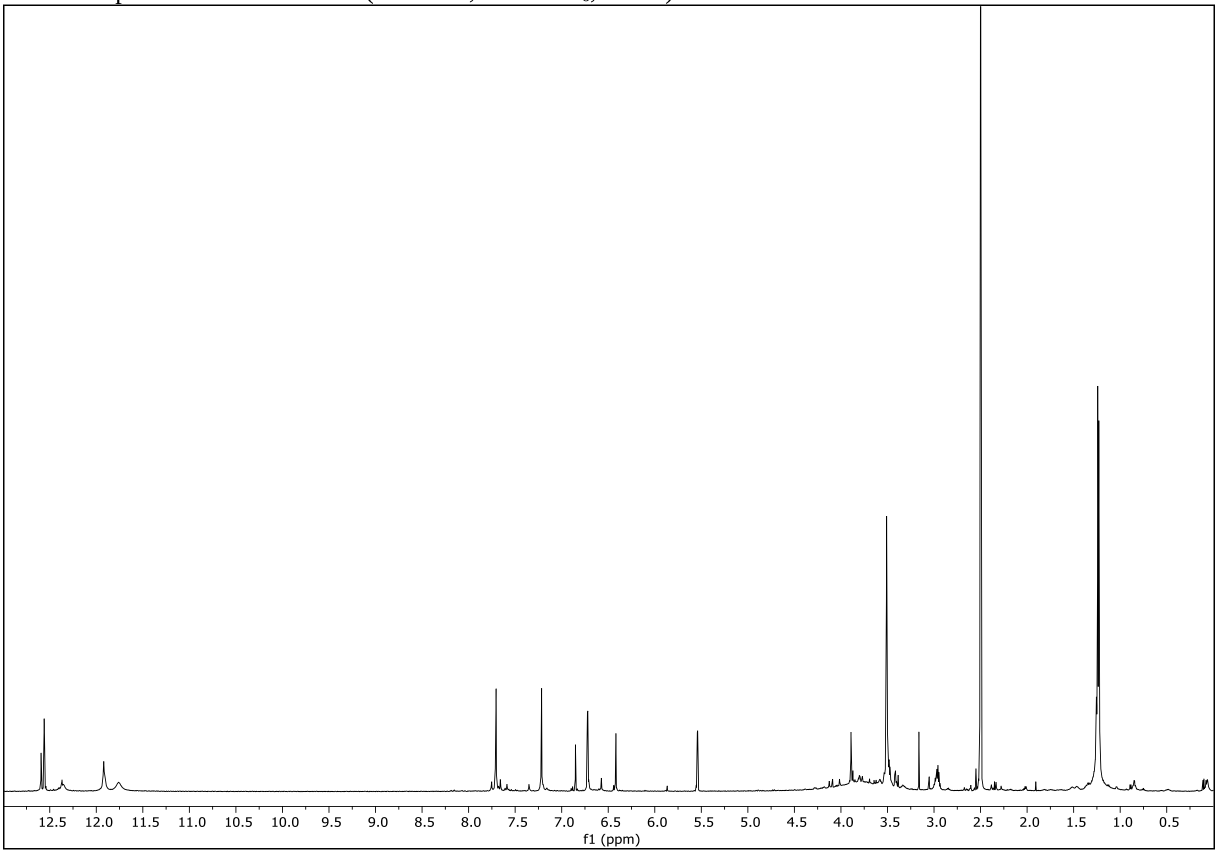

^{13}C NMR spectrum of RSH-O10b (150MHz, DMSO-d_6, 35 °C).

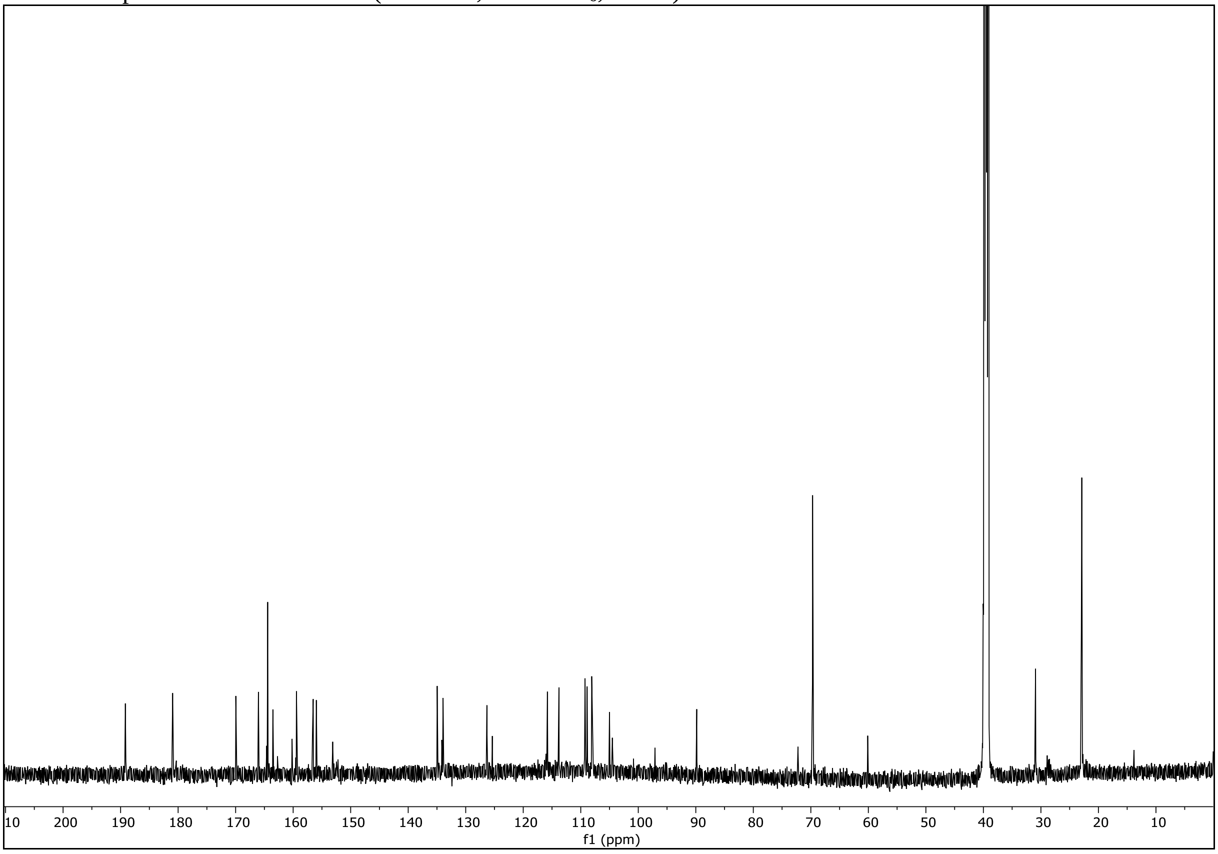

Figure S4e: NMR data of RSH-O3 (500/125MHz, DMSO-d_6, 25 °C)

Pos.	δ_C [ppm]	δ_H (*J* Hz) [ppm]	COSY	HMBC
1	164.0			2-H
2	88.0	5.10 d (1.6)	4-H	^1J, 4-H
3	171.4			2-H, 4-H, (6-H_2)
4	101.5	5.62 d (1.6)	2-H	2-H, 6-H_2
5	164.6			4-H, 6-H_2, (8-H)
6	36.6	3.64 s		4-H, ^1J, 8-H
7	134.4			4-H, 8-H, 9-H
8	120.8	6.70 d	9-H	6-H_2, ^1J, 9-H, 10-H
9	130.1	7.17 t (8.0)	8-H, 10-H	^1J
10	114.7	6.72 d	9-H	8-H, (9-H), ^1J
11	155.1			6-H_2, 9-H, 10-H
12	130.7			6-H_2, 9-H, 10-H
13	199.8			10-H, 16-H, (18-H)
14	119.3			16-H, 18-H, 20-H
15	158.9			16-H
16	99.9	6.04 d (2.2)	18-H	^1J, 18-H
17	160.7			16-H, 18-H
18	104.7	6.24 d (2.2)	16-H	16-H, ^1J, 20-H
19	151.9			20-H, 21-H_3, 22-H_3
20	29.1	3.00 hept (6.8)	21-H3, 22-H3	18-H, 21-H_3, 22-H_3
21	24.0	1.04 d (6.8)	20-H	20-H, ^1J, 22-H_3
22	24.0	1.04 d (6.8)	20-H	20-H, 21-H_3, ^1J
OH		10.21 br s<br>9.71 br s		

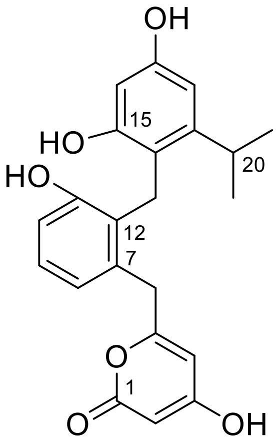

^{1}H NMR spectrum of RSH-O3 (500MHz, DMSO-d_6, 25 °C).

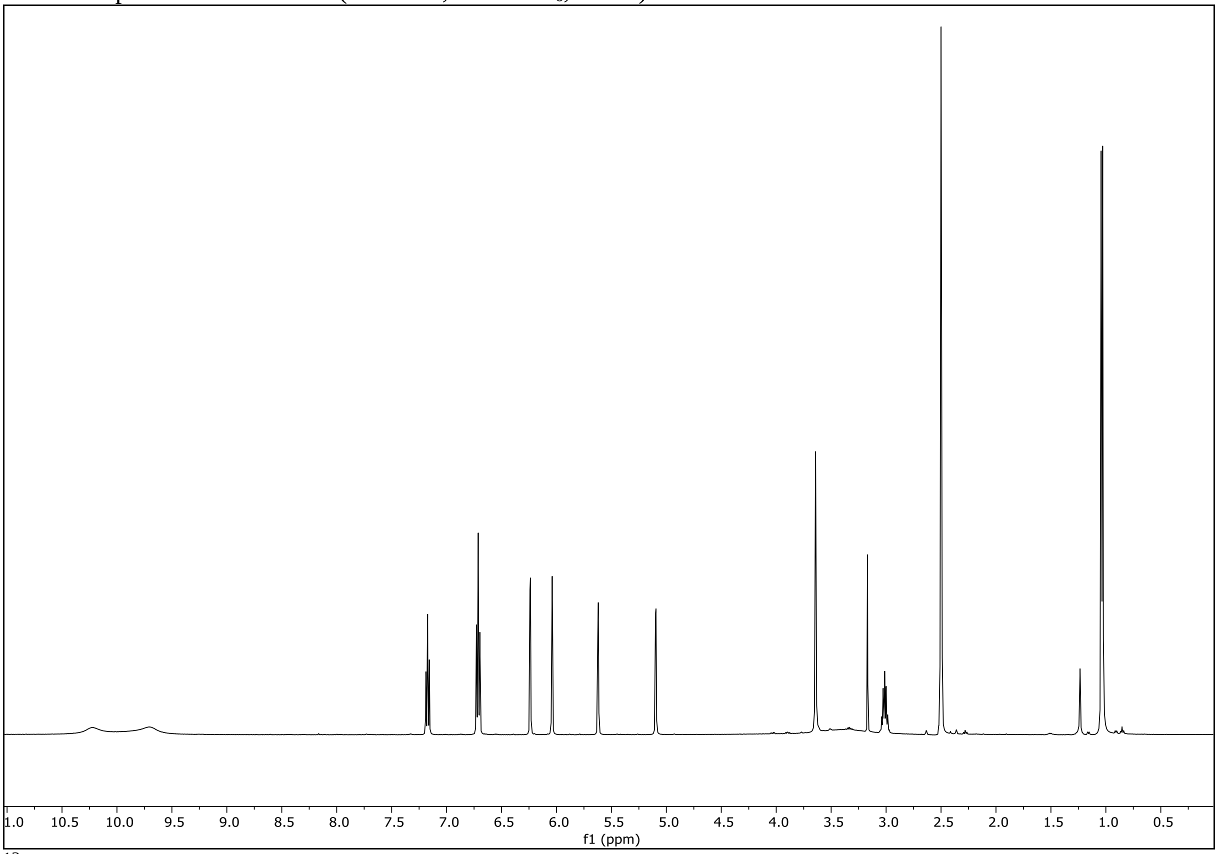

^{13}C NMR spectrum of RSH-O3 (125MHz, DMSO-d_6, 25 °C).

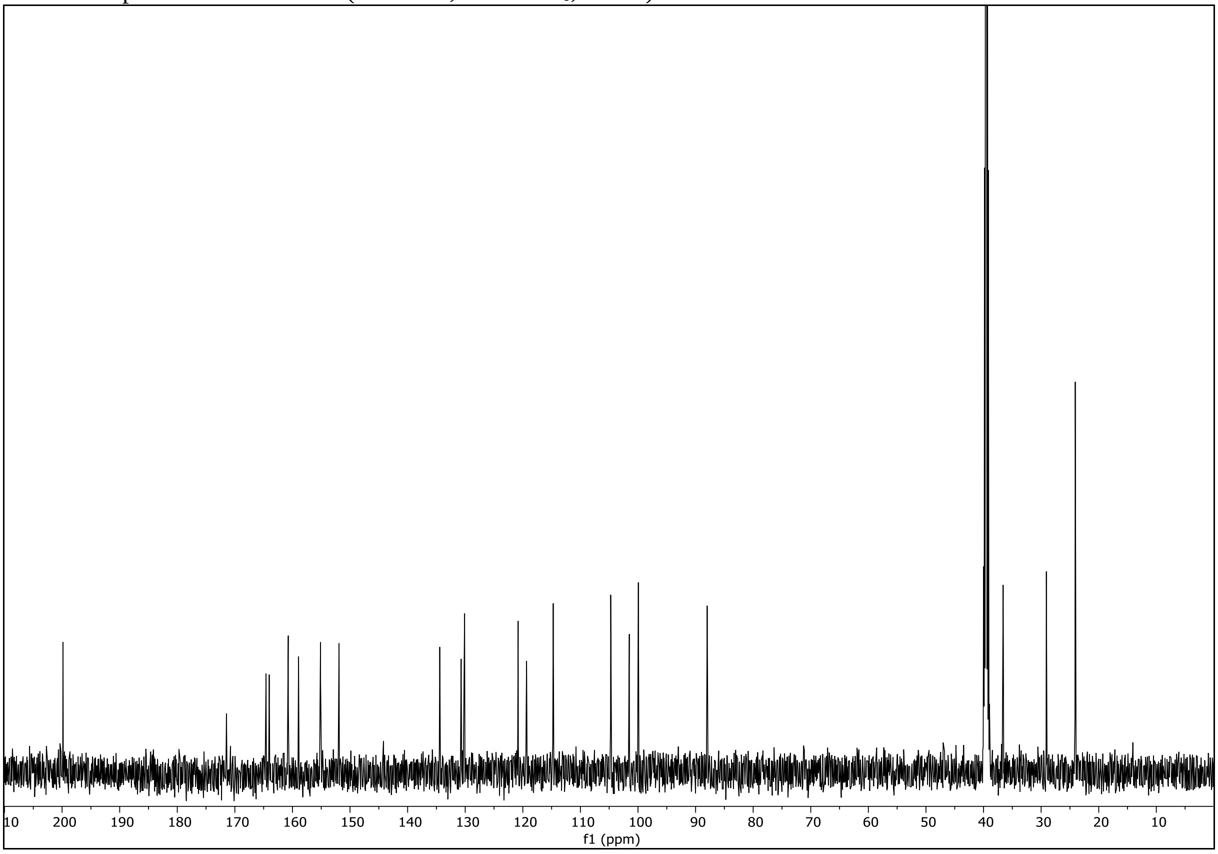

Figure S4f: NMR data of galvaquinone A (600/150MHz, DMSO-d_6, 35 °C)

Pos.	δ_C [ppm]	δ_H (J Hz) [ppm]	COSY	HMBC[a]
1	121.1	7.60 s	17-H_3	17-H_3
2	144.2			1-H, 17-H_3
3	136.0			1-H, 17-H_3
4	157.7			(1-H), 17-H_3
4a	114.2			1-H, 17-H_3
5	161.3			6-H, 7-H, (8-H)
6	124.4	7.39 dd (8.2, 1.0)	7-H, 8-H	(7-H), 8-H
7	137.5	7.83 dd (8.2, 7.4)	6-H, 8-H	^1J
8	119.4	7.72 dd (7.4, 1.0)	6-H, 7-H	6-H
8a	133.2			7-H
9	180.9			1-H, 8-H
9a	132.6			
10	191.5			(1-H, 8-H)
10a	115.8			6-H, (7-H), 8-H
11	205.2			12-H, 13-H_2
12	41.5	2.86 t (7.4)	13-H_2	13-H_2, 14-H
13	31.5	1.54 m	12-H_2, 14-H	12-H_2, 14-H_2, 15-H_3, 16-H_3
14	26.9	1.61 m	13-H_2, 15-H_3, 16-H_3	12-H_2, 13-H_2, 15-H_3, 16-H_3
15	22.2	0.90 d (6.8)	14-H	13-H_2, 14-H, 16-H_3
16	22.2	0.90 d (6.8)	14-H	13-H_2, 14-H, 15-H_3
17	19.2	2.31 s	1-H	1-H, ^1J
OH		11.94 br		

[a]weak signals in brackets

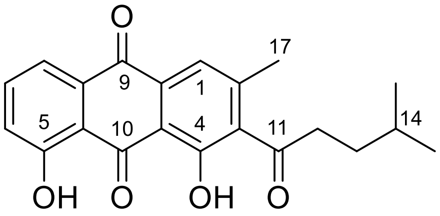

Figure S4g: NMR data of galvaquinone B (600/150MHz, Acetone-d_6, 25 °C)

Pos.	δ_C [ppm]	δ_H (J Hz) [ppm]	COSY	HMBC
1	157.8			17-H_3
2	137.1			17-H_3
3	144.1			17-H_3
4	[a]			
4a	112.0[b]			
5	163.3			7-H
6	125.5	7.40 dd (8.0, 1.2)	7-H	7-H, 8-H
7	138.3	7.88 dd (8.0, 7.6)	6-H, 8-H	6-H
8	120.2	7.90 dd (7.6, 1.2)	7-H	6-H
8a	134.4			7-H
9	187.3			8-H
9a	112.7[b]			
10	[a]			
10a	117.1			6-H, 8-H
11	204.7			12-H_2, 13-H_2
12	42.7	2.92 t (7.4)	13-H_2	13-H_2
13	32.6	1.62 m	12-H_2, 14-H	12-H_2, 14-H, 15-H_3, 16-H_3
14	28.3	1.67 m	13-H_2, 15-H_3, 16-H_3	12-H_2, 13-H_2, 15-H_3, 16-H_3
15	22.6	0.94 d (6.4)	14-H	14-H, 16-H_3
16	22.6	0.94 d (6.4)	14-H	14-H, 15-H_3
17	13.0	2.21 s		

[a]missing signal
[b]assignment not sure

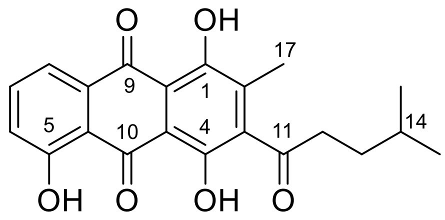

Figure 5: Plasmid maps used for the generation of constructis 1-6

Figure S5a: Plasmids used for the construction of *S. albus* J1074 x pUWLR1R2R3 x construct 1 and *S. albus* J1074 x pUWLR1R2R3 x construct 2.

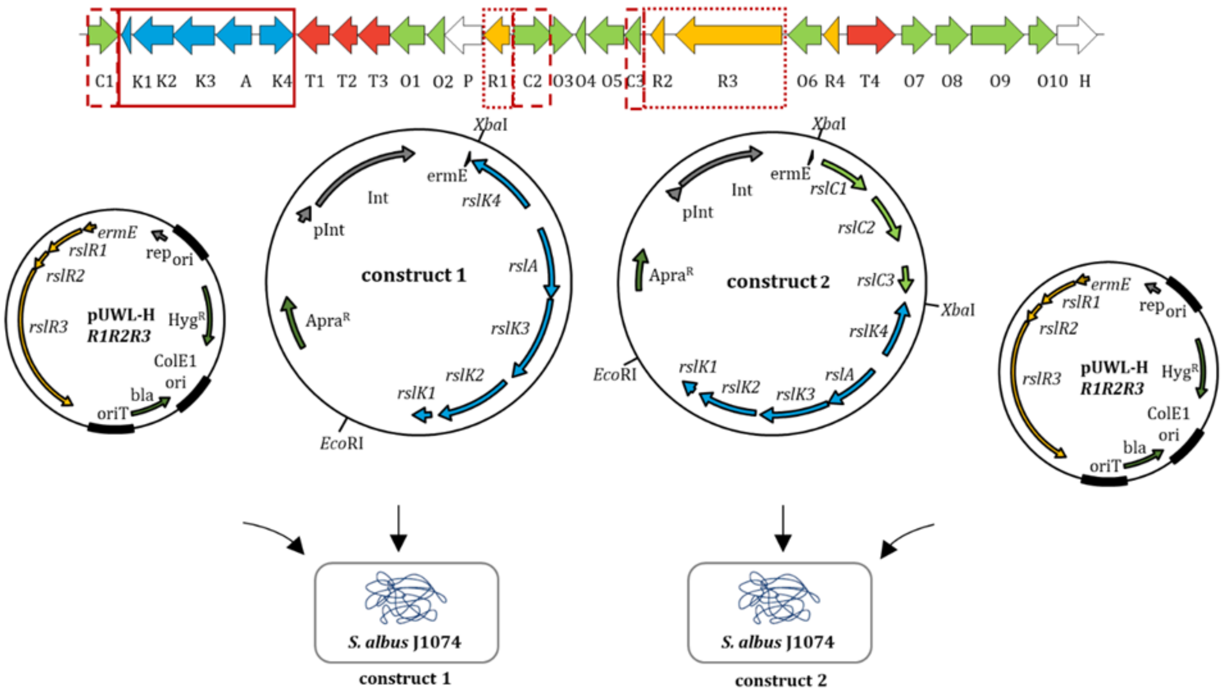

Figure S5b: Plasmids used for the construction of *S. albus* J1074 x pUWLR1R2R3 x construct 3 and *S. albus* J1074 x pUWLR1R2R3 x construct 4. The gene *rslO10* was integrated via pTOSz.

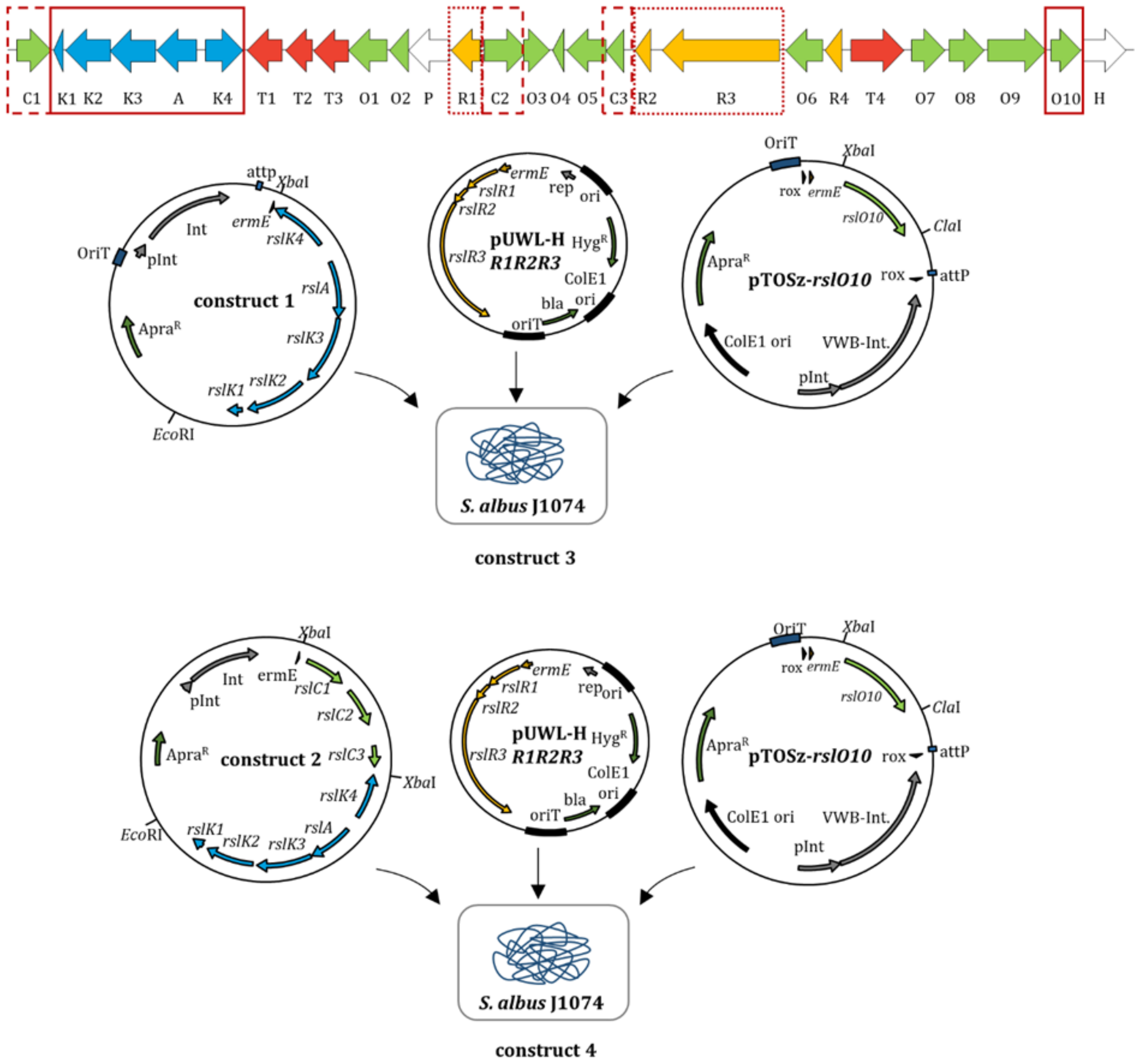

Figure S5c: Plasmids used for the construction of *S. albus* J1074 x pUWLR1R2R3 x construct 5 and *S. albus* J1074 x pUWLR1R2R3 x construct 6. The genes *rslO10* and *rslO3* were integrated via pTOSz.

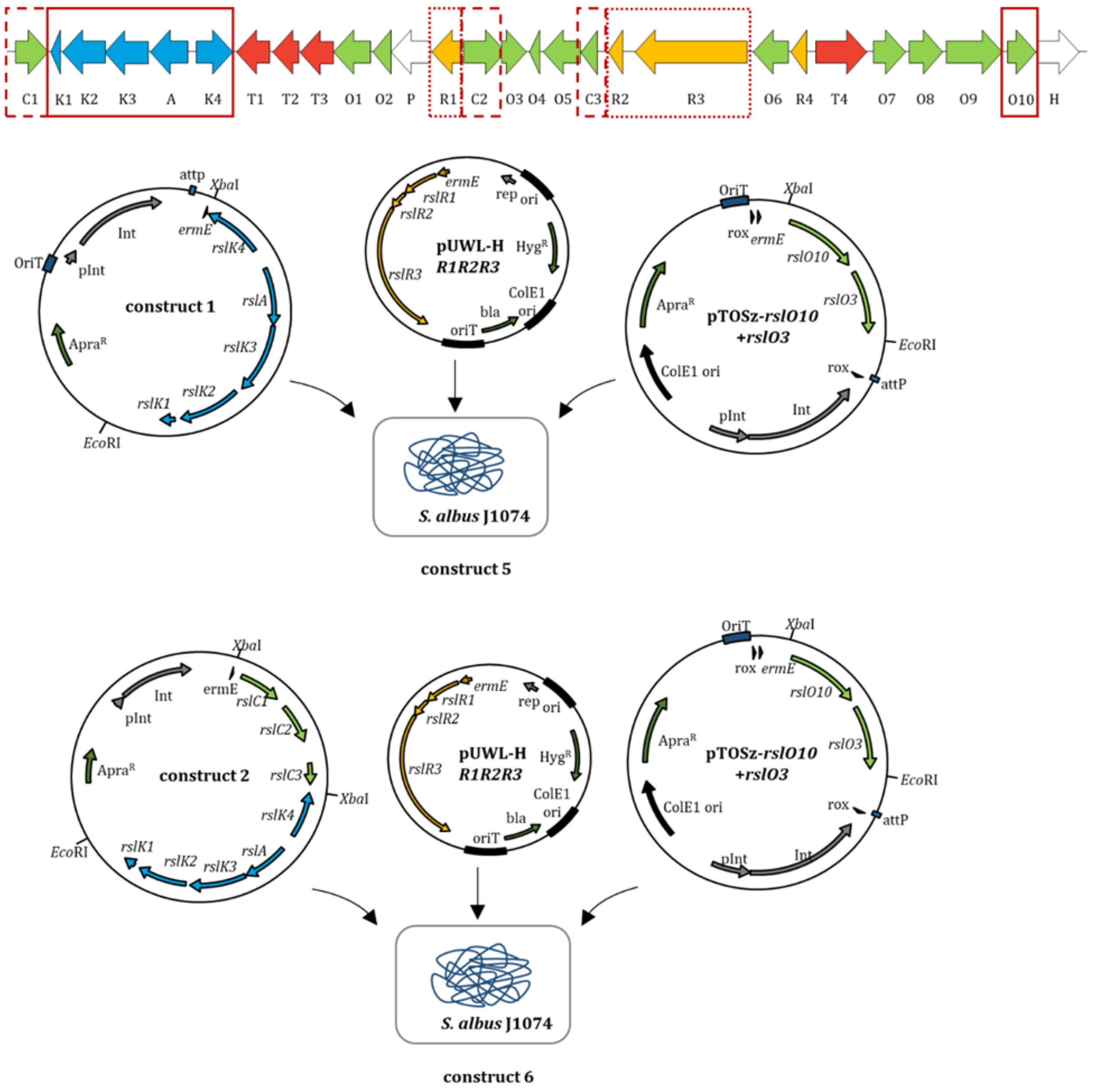

Supplement: Supplementary file 1 [file molecules-25-01955-s001.pdf]
